# Dehydrocostus Lactone Effectively Alleviates Inflammatory Diseases by Covalently and Irreversibly Targeting NLRP3

**DOI:** 10.1002/mco2.70367

**Published:** 2025-09-03

**Authors:** Qi Lv, Yishu Zhang, Juan Wang, Weijiang Lin, Ying Xie, Hongqiong Yang, Xunkai Yin, Zhenzhen Zhu, Yifan Cui, Yang Hu, Li Zeng, Yinan Zhang, Xubing Chen, Jian Liu, Lihong Hu

**Affiliations:** ^1^ Jiangsu Key Laboratory of Functional Substance of Chinese Medicine School of Pharmacy Nanjing University of Chinese Medicine Nanjing China; ^2^ State Key Laboratory of Quality Research in Chinese Medicines Faculty of Chinese Medicine Macau University of Science and Technology Macau China; ^3^ College of Pharmaceutical Science Dali University Dali China

**Keywords:** covalent irreversible, cysteine 280, dehydrocostus lactone, NLRP3 inflammasome, NLRP3‐driven diseases

## Abstract

The activation of nucleotide oligomerization domain‐like receptor (NLR) family, pyrin domain‐containing protein 3 (NLRP3) inflammasome is implicated in the pathogenesis of various inflammatory diseases. The natural product oridonin possesses a novel mechanism for NLRP3 inhibition and a unique binding mode with NLRP3, but its poor anti‐inflammatory activity limits further application. After virtual screening of diverse natural product libraries, dehydrocostus lactone (DCL) was considered as a potential NLRP3 inhibitor. DCL effectively inhibited caspase‐1 cleavage and release of IL‐1β in mouse and human macrophages at an extremely low concentration of 10 nM, comparable to MCC950. Mechanistically, our study assigned DCL a novel role in disrupting NLRP3 inflammasome assembly and ASC oligomerization. Excluding the influence on potassium/chloride ion efflux, calcium ion influx, and production of mitochondrial ROS, DCL formed a covalent bond with cysteine 280 in NACHT domain of NLRP3, thereby inhibiting the interaction between NLRP3 and NEK7. Furthermore, DCL exhibited protective effects in mouse models of NLRP3 inflammasome‐mediated diseases, including dextran sulfate sodium‐induced colitis, 2,4,6‐trinitrobenzenesulfonic acid‐induced Crohn's disease, LPS‐induced septic shock, and monosodium urate‐induced peritonitis. Our findings identify NLRP3 as the direct target of DCL, positioning DCL as a promising lead compound for treatment of NLRP3 inflammasome‐related diseases.

## Graphical abstract

Dehydrocostus lactone (DCL) covalently binds to NLRP3 and effectively reduces the interaction between NEK7 and NLRP3, thereby blocking the assembly of NLRP3 inflammasome complex and subsequent activation in macrophages. Consequently, DCL inhibits the cleavage and maturation of pro‐IL‐1β and pro‐IL‐18, ultimately leading to the suppression of the inflammatory response.

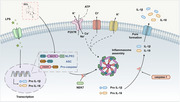

## 1 Introduction

Moderate inflammatory response is essential for defending against infections and tissue damage, but excessive inflammation can lead to the immune dysfunction [1]. Inflammasomes are critical components of the innate immune system, with the NLRP3 inflammasome serving as the cornerstone of the innate immune response. NLRP3, the most extensively studied member of the NLR family, can recognize various pathogen‐associated molecular patterns (PAMPs) and danger‐associated molecular patterns (DAMPs), triggering the assembly of inflammasome complex composing NLRP3, pro‐caspase‐1, and adaptor protein apoptosis‐associated speck‐like protein containing a caspase recruitment domain (ASC) [2]. This assembly will induce the formation of activate caspase‐1, which cleaves gasdermin D to initiate pyroptosis and facilitates the maturation of IL‐1β and IL‐18, contributing to the development of inflammatory diseases including colitis [3], cryopyrin‐associated periodic syndrome [4], type 2 diabetes (T2D) [5], gout [6], atherosclerosis [7], and peritonitis [8]. Consequently, targeting NLRP3 inflammasome activation represents a promising therapeutic strategy for inflammatory diseases.

Recently, the discovery and development of NLRP3 inflammasome inhibitors have attracted considerable attention, with several promising inhibitors being reported. Tranilast and CY‐09 directly bind to the ATP‐binding motif of NLRP3 NACHT domain, inhibiting ATPase activity and thereby suppressing NLRP3 assembly to mitigate inflammatory diseases [9, 10]. MCC950, a diarylsulfonylurea compound, is one of the most potent and selective NLRP3 inhibitors, binding to the Walker B motif of NACHT domain to promote a closed and inactive conformation of NLRP3 [11, 12]. This inhibition demonstrates favorable pharmacodynamic effects on diseases such as atherosclerosis, Alzheimer's disease, colitis, diabetes, etc. [13, 14]. Despite advancing to phase II clinical trials for rheumatoid arthritis, MCC950 was discontinued due to drug‐induced liver injury [11]. Efforts to optimize MCC950's structure have led to the development of other sulfonylurea derivatives such as ZYIL1, IZD‐174, and RG‐6418, which have entered clinical trials [15]. Nonetheless, the underlying cause of MCC950‐induced liver injury remains unclear, and the potential liver toxicity of these derivatives cannot be ruled out. Besides, these derivatives exhibit poor dose dependence, with curative effects saturating at high dosages. Consequently, the exploration of other NLRP3 inflammasome inhibitors with novel binding mechanisms had garnered significant interests.

Oridonin (Ori), a natural bioactive ent‐kaurane diterpenoid, is the primary component of *Rabdosia rubescens*. In 2018, Zhou's research group confirmed that Ori formed a covalent bond with Cys279 of NLRP3, thereby inhibiting inflammasome assembly and activation [6]. Interestingly, the binding site of Ori to NLRP3 was completely different from that of MCC950, as Cys279 was not located at the ATP‐binding site of NLRP3. Thus, Ori offered a novel mechanism for NLRP3 inhibition and a unique binding mode with NLRP3, potentially addressing the MCC950‐induced liver injury. However, the inhibitory activity of Ori against NLRP3 was moderate, highlighting the need to identify novel natural products with stronger inhibitory activity against NLRP3.

In this study, we screened diverse natural product libraries and identified several natural products as NLRP3 inhibitors. Among them, dehydrocostus lactone (DCL) specifically halted the activation of NLRP3 inflammasome in human and mouse macrophages at an extremely low concentration of 10 nM. Furthermore, DCL was the first to be reported to covalently and irreversibly bound to Cys280 in the NACHT domain of NLRP3, disrupting the association between NEK7 and NLRP3. This prevented the assembly and activation of NLRP3 inflammasome, distinguishing DCL from MCC950. More importantly, DCL significantly relieved colon inflammation, LPS‐induced septic shock, and monosodium urate (MSU)‐induced peritonitis in mice at an extremely low dosage (i.p. 5 µg/kg/day) in vivo. Therefore, DCL serves as a potent and specific NLRP3 inhibitor, offering promise as a therapeutic agent for a wide range of NLRP3 inflammasome‐mediated diseases.

## 2 Results

### 2.1 Virtual Screening of Potential NLRP3 Inhibitors From Natural Product Libraries

**FIGURE 1:**
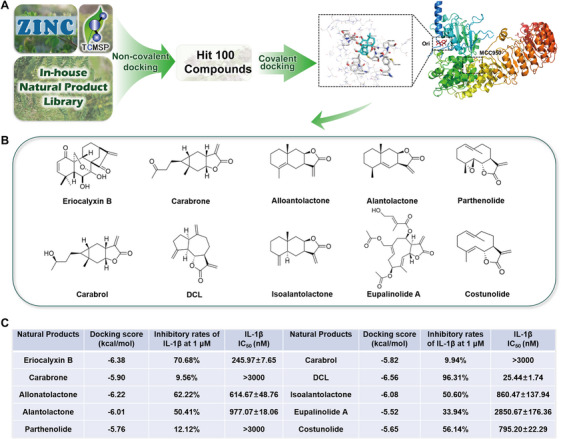
Comprehensive screening process of NLRP3 inhibitors. (A) The screening process of discovering potential NLRP3 inhibitors from TCM database. The figure was created with BioRender.com. (B) Ten potential NLRP3 inhibitors were finally screened out and their structures were exhibited. (C) The IL‐1β inhibition rate at 1 µM and corresponding IC_50_ values in BMDMs for the 10 natural products with top‐ranked molecular docking scores.

Oridonin has been shown to inhibit NLRP3 inflammasome activation primarily through covalent binding to Cys279 of NLRP3 [6]. To identify additional potential inhibitors, we conducted a virtual screening of integrated natural product libraries (ZINC, TCMSP, and our in‐house natural product library). These compounds were docked into the binding pocket surrounding Cys279 within a 20Å radius in a non‐covalent mode. The top 100 hits with the highest docking scores were selected for subsequent covalent docking calculations (Figure 1A). Given the highly nucleophilicity of cystine and the prevalence of covalent drugs interacting with cysteine residues, we performed covalent docking studies. The covalent reaction residue was set as cysteines, including Cys261, Cys279, Cys280, Cys319, and Cys514, and ligands at the binding pocket were calculated. Meanwhile, the setting of covalent reaction types was based on specific covalent warheads; for instance, natural products containing a special α,β‐unsaturated carbonyl group were set for Michael addition reactions. Compounds lacking these covalent warheads were excluded from further analysis. Ultimately, the top 10 natural products with the most favorable covalent docking scores and strong covalent interaction with NLRP3 were selected for further assessment of their NLRP3 inhibitory activity (Figure 1B). The Western blotting analysis revealed that DCL exhibited the most potent inhibitory effect on the protein expression of cleaved IL‐1β and cleaved caspase‐1 at the concentration of 1 µM (Figure S1). Consistently, ELISA demonstrated that DCL exhibited the highest inhibition rate on IL‐1β levels among all tested compounds (96%, Figure 1C). To more precisely characterize the inhibitory potency of each compound, we determined their IC_50_ values for IL‐1β suppression. Similarly, DCL showed the lowest IC_50_ value of 25.44 nM, and was therefore selected for further biological evaluation.

### 2.2 DCL Selectively Suppresses Inappropriate Activation of NLRP3 Inflammasome in Macrophages

**FIGURE 2:**
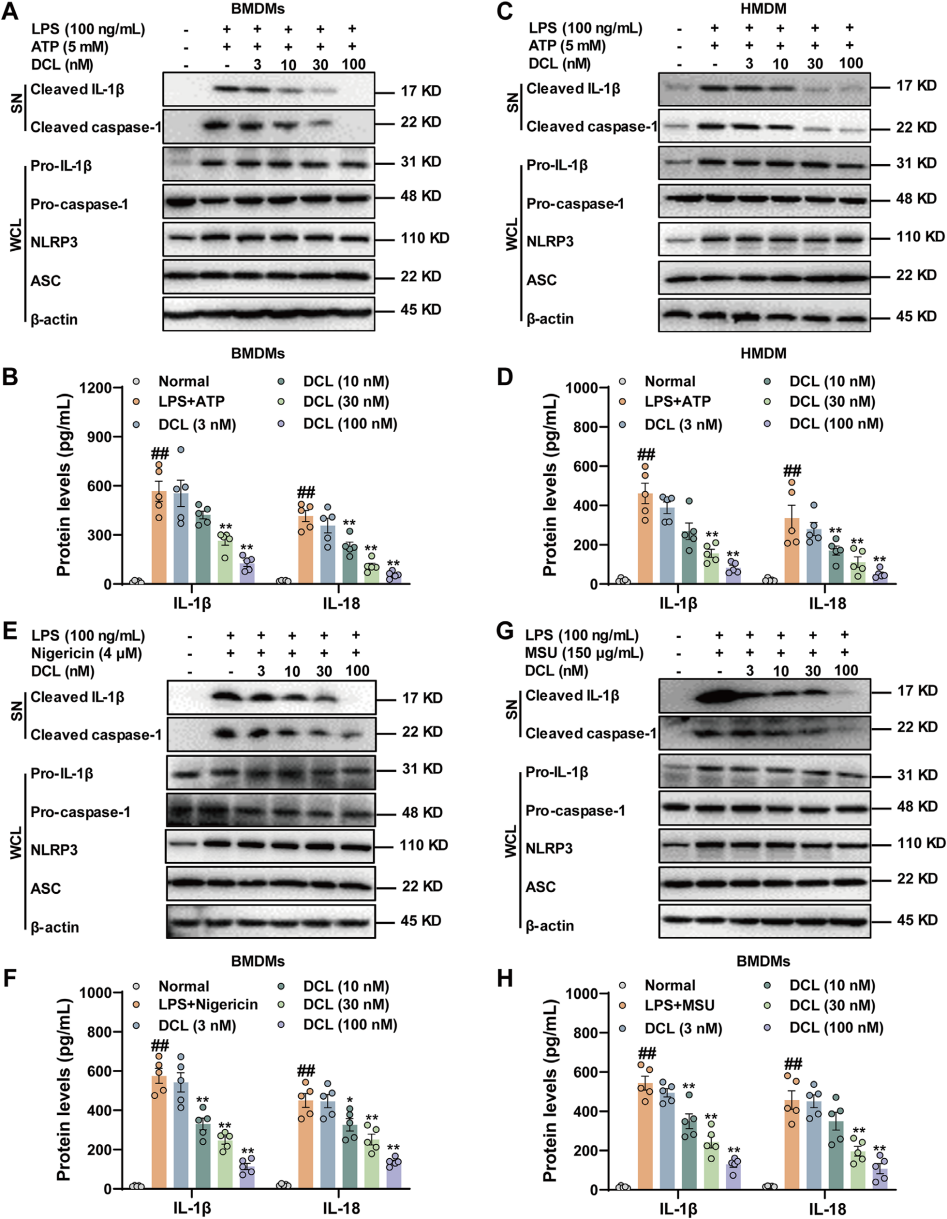
DCL specifically inhibits NLRP3 inflammasome activation. (A and C) Western blotting analysis of cleaved caspase‐1 and cleaved IL‐1β in supernatant of BMDMs and HMDMs treated with LPS (100 ng/mL, 4 h) and ATP (5 mM, 45 min) ± DCL (3, 10, 30, and 100 nM, 1 h). (B and D) ELISA analysis of IL‐1β and IL‐18 in supernatant of BMDMs and HMDMs treated with LPS (100 ng/mL, 4 h) and ATP (5 mM, 45 min) ± DCL (3, 10, 30, and 100 nM, 1 h). (E and G) Western blotting analysis of cleaved caspase‐1 and cleaved IL‐1β in supernatant of BMDMs treated with LPS (100 ng/mL, 4 h) and nigericin (4 µM, 1 h) or MSU (150 µg/mL, 6 h) ± DCL (3, 10, 30, and 100 nM, 1 h). (F and H) ELISA analysis of IL‐1β and IL‐18 in supernatant of BMDMs treated with LPS (100 ng/mL, 4 h) and nigericin (4 µM, 1 h) or MSU (150 µg/mL, 6 h) ± DCL (3, 10, 30, and 100 nM, 1 h). Data from in vitro assays were presentative of at least three independent experiments. Values are shown as means ± S.E.M. ^#^ *p* < 0.05 and ^##^ *p* < 0.01 versus normal group. ^*^ *p* < 0.05 and ^**^ *p* < 0.01 versus LPS + ATP, MSU, or nigericin group.

To explore the potential role of DCL in inhibiting NLRP3 inflammasome activation in vitro. The BMDMs were first primed with LPS and then treated with DCL (3, 10, 30, and 100 nM) for 1 h, followed by stimulation with ATP for 45 min. As anticipated, DCL (10, 30, and 100 nM) markedly inhibited pro‐caspase‐1 cleavage and pro‐IL‐1β cleavage into biologically active form (Figure 2A). Meanwhile, the production of IL‐1β and IL‐18 was greatly weakened by DCL at the indicated concentration (Figure 2B). Furthermore, to rule out the possibility that the above‐mentioned phenomenon can only be observed in primary mouse macrophages, the inhibition efficacy of DCL on NLRP3 inflammasome activation was also determined in human peripheral blood mononuclear cell‐derived macrophages (HMDMs). Interestingly, similar findings including impaired expression of cleaved caspase‐1 and IL‐1β, and release of IL‐1β and IL‐18 were achieved in HMDMs in the presence of DCL (Figure 2C,D). Meanwhile, DCL (3, 10, 30, and 100 nM) showed no effect on proliferation, viability, and apoptosis of BMDMs and HMDM (Figure S2A‒F). These data indicate that DCL profoundly suppresses NLRP3 inflammasome activation in both human and murine macrophages.

Additionally, we stimulated NLRP3 inflammasome in BMDMs with LPS and nigericin/MSU. Consistently, DCL (10, 30, and 100 nM) also apparently impaired nigericin or MSU‐induced cleavage of pro‐IL‐1β and pro‐caspase‐1, thereby reducing secretion of IL‐1β in BMDMs. In addition, DCL diminished nigericin‐induced IL‐18 generation at concentrations of 10, 30, 100 nM, and attenuated MSU‐induced IL‐18 production at 10, 100 nM (Figure 2E‒H). More importantly, DCL showed no influence on poly(dA:dT)‐induced AIM2 inflammasome, bacterial flagellin‐induced NLRC4 inflammasome, or muramyl dipeptide (MDP)‐induced NLRP1 inflammasome activation, as manifested by unchanged expression of cleaved caspase‐1 and cleaved IL‐1β (Figure S3A‒D). To summarize, these findings demonstrate that DCL‐mediated inhibitory effect is specific to the NLRP3 inflammasome.

### 2.3 DCL Blocks Assembly Stage of NLRP3 Inflammasome Activation

**FIGURE 3:**
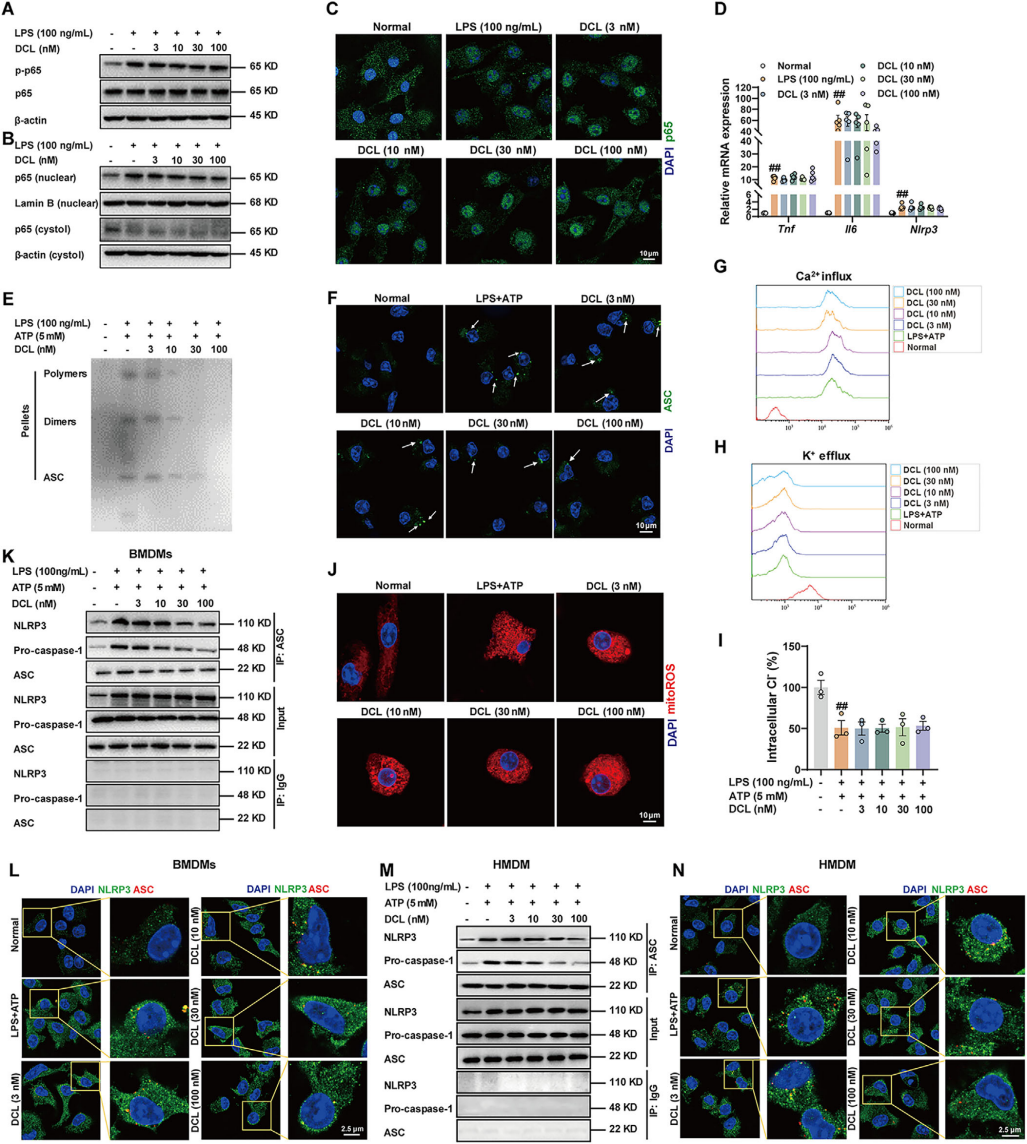
DCL inhibits assembly stage of NLRP3 inflammasome activation. (A) Western blotting analysis of p65 phosphorylation modification in BMDMs treated with DCL (3, 10, 30, and 100 nM, 4 h) ± LPS (100 ng/mL, 30 min). (B and C) Western blotting and confocal analysis of p65 nuclear translocation in BMDMs treated with DCL (3, 10, 30, and 100 nM, 4 h) ± LPS (100 ng/mL, 4 h). (D) qPCR analysis of *Nlrp3*, *Il6*, and *Tnf* in BMDMs treated with DCL (3, 10, 30, and 100 nM, 4 h) ± LPS (100 ng/mL, 4 h). (E‒M) BMDMs or HMDMs were subjected to the following treatment: LPS priming (100 ng/mL, 4 h), DCL (3, 10, 30, and 100 nM, 1 h), and ATP (5 mM, 45 min). The Western blotting analysis of ASC oligomerization (E); laser scanning confocal microscopy of ASC speck (F); flow cytometry analysis of K^+^ and Ca^2+^ content (G and H); the intracellular Cl^−^ concentration was detected by MQAE (I); the immunofluorescence analysis of mitochondrial ROS (J); the co‐immunoprecipitation analysis of NLRP/ASC/pro‐caspase‐1 complex (K and M); confocal microscopy analysis of co‐localization between NLRP3 and ASC (L and N). Data from in vitro assays were presentative of at least three independent experiments. Values are shown as means ± S.E.M. ^#^ *p* < 0.05 and ^##^ *p* < 0.01 versus normal group.

We next explored the potential mechanism by which DCL inhibited NLRP3 inflammasome activation in macrophages. The canonical activation of NLRP3 inflammasome requires two steps: priming and activation. The priming step is mainly provided by LPS, which induces the NF‐κB signaling transduction and subsequent transcription of *Nlrp3*, *Il18*, and *Il1* gene, providing material basis for the activation of NLRP3 inflammasome. The activation step is primarily triggered by PAMPs and DAMPs, which enhances NLRP3 inflammasome assembly, subsequent caspase‐1 activation and ultimately IL‐1β and IL‐18 maturation [16]. DCL (3, 10, 30, and 100 nM) exerted little effect on LPS‐induced NF‐κB signaling pathway activation, evidenced by remained phosphorylation modification of p65 (Figure 3A). Similarly, the western blotting and confocal microscopy results showed no discernible change in p65 nuclear translocation at different concentrations of DCL in BMDMs (Figure 3B,C). Meanwhile, the mRNA expression of *Nlrp3*, *Il6*, and *Tnf* showed no variances in BMDMs treated with DCL and LPS (Figure 3D), and the primer sequences used for these genes were provided in Table S1. These data clearly suggest that DCL‐mediated NLRP3 inflammasome inactivation in macrophages is independent of the priming step.

ASC, an adapter protein can aggregate into macromolecules dimers, namely ASC speck, which is pivotal for caspase‐1 activation and considered a credible marker of NLRP3 inflammasome activation [17]. Therefore, we evaluated the influence of DCL on ASC speck formation during the process of NLRP3 inflammasome activation. Accordingly, DCL (10, 30, and 100 nM) curbed the ASC speck formation and ASC oligomerization in BMDMs (Figure 3E,F), clearly suggesting that upstream of ASC oligomerization was critical for DCL‐blunted NLRP3 inflammasome activation. Unexpectedly, DCL (10, 30, and 100 nM) had no discernible effect on upstream events of NLRP3 inflammasome activation, including mitochondria ROS generation, Ca^2+^ influx, K^+^ efflux, or Cl^−^ efflux (Figure 3G‒J). To further confirm that DCL inhibited NLRP3 inflammasome activation independently of K^+^ efflux, we established an alternative activation model using LPS and imiquimod, which stimulated the TLR7/MyD88 pathway, ROS generation, and bypassing the conventional potassium efflux‐dependent mechanism. As shown in Figure S4, DCL retained its inhibitory activity in this context, as evidenced by the reduced protein expression of cleaved caspase‐1 and cleaved IL‐1β.

Lastly, the impact of DCL on NLRP3 inflammasome assembly was tested. Co‐immunoprecipitation (Co‐IP) analysis showed that DCL (10, 30, and 100 nM) robustly blunted the formation of NLRP3/pro‐caspase‐1/ASC complex triggered by LPS plus ATP (Figure 3K). Similarly, the confocal microscopy results revealed an inhibitory effect of DCL on co‐localization between NLRP3 and ASC (Figure 3L). Notably, this NLRP3/ASC/pro‐caspase‐1 interaction was substantially compromised by DCL in the HMDMs (Figure 3M,N). These results strongly confirm that DCL blocks NLRP3 inflammasome activation through disrupting the assembly process.

### 2.4 DCL Directly Binds to NLRP3

**FIGURE 4:**
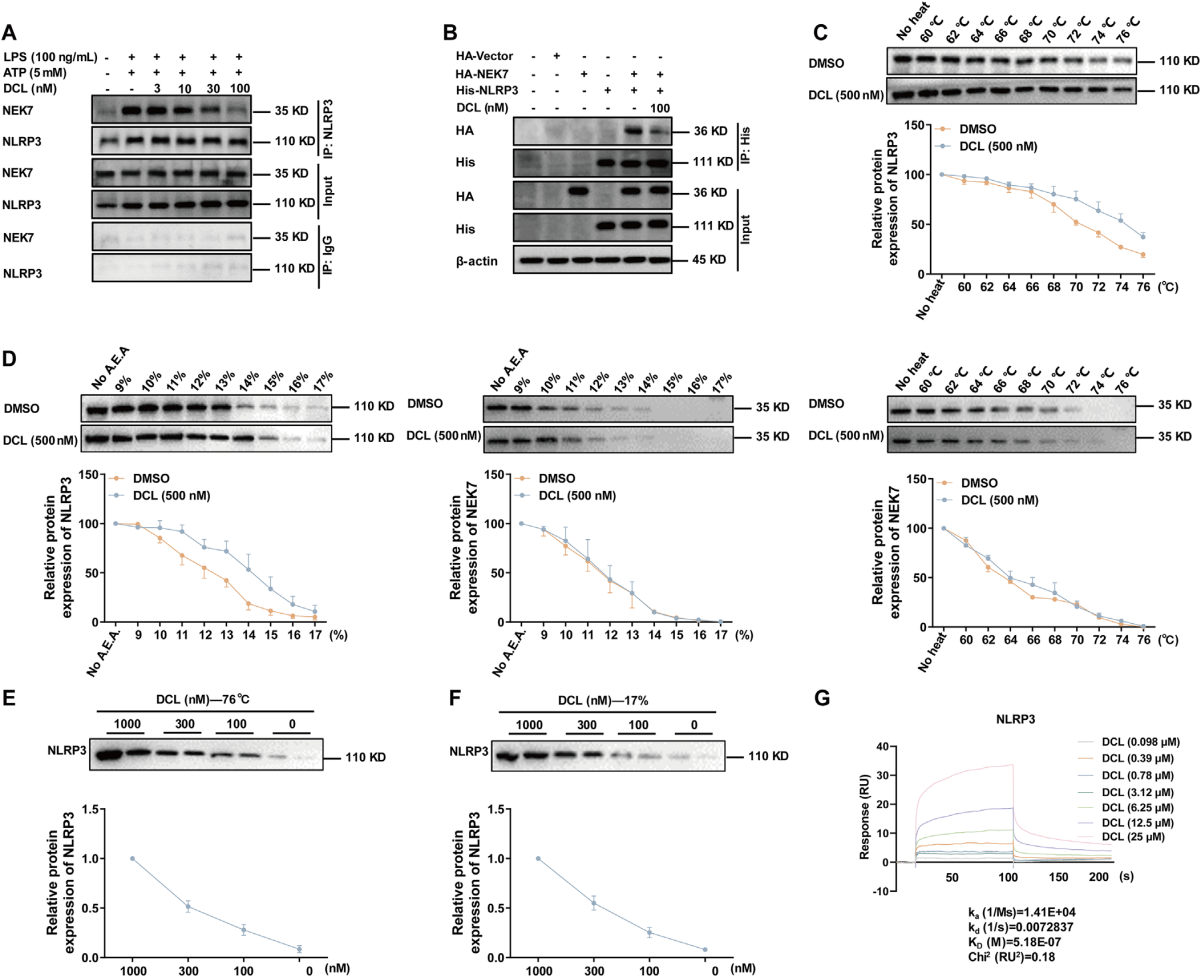
DCL directly binds to NLRP3. (A) BMDMs were subjected to the following treatment: LPS priming (100 ng/mL, 4 h), DCL (3, 10, 30, and 100 nM, 1 h), and ATP (5 mM, 45 min). The co‐immunoprecipitation analysis of NEK7/NLRP3 complex. (B) The HEK‐293T cells were transfected with plasmids (HA‐NEK7, His‐NLRP3) in six‐well plates through the Lipofectamine 3000. After treatment with DCL (100 nM) for 24 h, the interaction between NEK7 and NLRP3 was detected by co‐immunoprecipitation analysis. (C and D) The cell lysates of BMDMs were incubated with DCL (500 nM) for 1 h and then subjected to different temperature or different proportions of organic solvents. Western blotting analysis of NLRP3 and NEK7 thermal/chemical stability. (E and F) The cell lysates of BMDMs were incubated with DCL (0, 100, 300, and 1000 nM) for 1 h and then subjected to 76°C or the constant ratio of organic solvent of 17%. Western blotting analysis of NLRP3 and NEK7 thermal/chemical stability. (G) SPR analysis of direct association between NLRP3 and DCL. (H) The ATPase activity of NLRP3 was determined by kits. Data from in vitro assays were presentative of at least of three independent experiments. Values are shown as means ± S.E.M.

During the activation phase of NLRP3 inflammasome, the association between NEK7 and NLRP3 mediates inflammasome assembly process [18]. Therefore, we proposed the hypothesis that DCL might prevent inflammasome assembly through decreasing the interaction between NLRP3 and NEK7. As anticipated, the results of Co‐IP showed that DCL (10, 30, and 100 nM) reduced the binding between NEK7 and NLRP3 in a concentration‐dependent fashion (Figure 4A). To further validate the effect of DCL on NEK7/NLRP3 complex formation, we constructed the corresponding plasmids and co‐transfected them into the HEK‐293T cells to examine the interaction in a controlled cellular system. The additional experiments confirmed that DCL markedly inhibited the formation of NEK7/NLRP3 complex (Figure 4B), suggesting that DCL might directly target either NLRP3 or NEK7 to decrease their interaction.

To identify the potential molecular target of DCL, we designed and synthesized DCL‐P, a chemical probe derived from the parent compound. Our results demonstrated that DCL‐P retained comparable inhibitory activity against NLRP3 inflammasome as DCL itself (Figure S5A,B). When the cell lysates from BMDMs were incubated with a synthesized biotinylated DCL (DCL‐P), NLRP3, but not NEK7, was pulled down (Figure S5C). These data further suggested that DCL might bind and sequester NLRP3 protein, thereby preventing formation of NEK7‒NLRP3 complex. To more directly confirm that DCL directly bound to NLRP3 rather than NEK7, we performed the cellular thermal shift assay and solvent‐induced protein precipitation (SIP) assays. As shown in Figure 4C, compared to the DMSO control, DCL enhanced the thermal stability of NLRP3 by 4.0°C in BMDMs. On the contrary, DCL exerted non‐existent effect on the thermal stability of NEK7. Consistently, the association between DCL and NLRP3 protein was validated by SIP analysis (Figure 4D). Additionally, the cells were treated with four different concentrations of DCL and then acted for 3 min at a constant temperature of 76°C or a constant proportion of chemical reagent of 17%. The results in Figure 4E,F indicated that DCL enhanced the stability of NLRP3 in a concentration‐dependent manner. In order to more precisely quantify the association between DCL and the purified NLRP3 protein, the surface plasmon resonance assay was performed. The affinity constant (*K*_D_) of DCL‒NLRP3 was around 518 nM (Figure 4G). Collectively, these data suggest that DCL directly interacts with NLRP3, thus disrupting NEK7‒NLRP3 interaction and the subsequent inflammasome assembly.

### 2.5 DCL Targets Cys280 of NLRP3 Via Covalent Bond Formation

**FIGURE 5:**
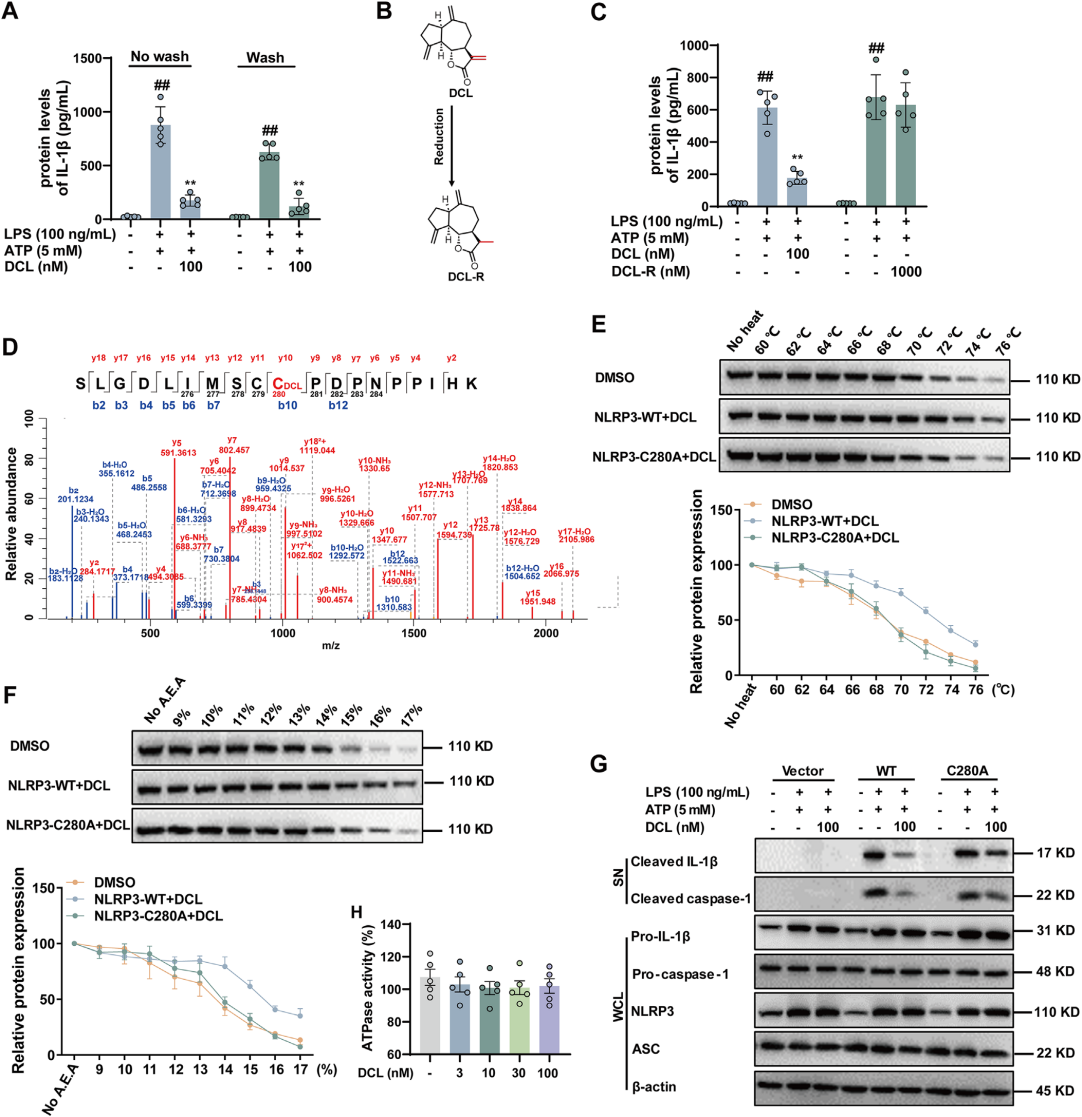
DCL targets Cys280 of NLRP3 via covalent bond formation. (A) BMDMs were primed with LPS (100 ng/mL, 4 h) and then treated with DCL (100 nM) for 30 min. Subsequently, the cells undergo extensive PBS for 30 min to remove extracellular, unbound DCL before being stimulated with ATP (5 mM, 45 min). The release of IL‐1β was assessed by ELISA. (B) Structure of DCL without carbon‒carbon double‐bond (DCL‐R). (C) ELISA analysis of IL‐1β in supernatant of BMDMs treated with LPS (100 ng/mL, 4 h) and ATP (5 mM, 45 min) ± DCL (100 nM, 1 h) or DCL‐R (1000 nM, 1 h). (D) Mass spectrogram of the modified peptide in exogenous NLRP3 treated with DCL. (E and F) Cells were transfected with WT or mutant NLRP3 construct (C280A). Subsequently, the cells were lysed and then treated with DCL (500 nM) for 1 h, followed by treatment with different temperature and different proportion of organic solvent. Western blotting analysis of NLRP3 stability. (G) Western blotting analysis of the cleaved IL‐1β and activated caspase‐1 or ELISA of IL‐1β in supernatants from LPS‐primed *Nlrp3* ^−/−^ BMDMs reconstituted with WT or mutant NLRP3 (C280A) that were pretreated with DCL (100 nM) for 1 h and then stimulated with ATP for 45 min. Western blotting analysis of cleaved caspase‐1 and cleaved IL‐1β in supernatant. Data from in vitro assays were presentative of at least of three independent experiments. Values are shown as means ± S.E.M. ^#^ *p* < 0.05 and ^##^ *p* < 0.01 versus normal group. ^**^ *p* < 0.01 versus LPS + ATP group.

To gain mechanistic insight of DCL interaction with NLRP3, we first determined whether the inactivation of NLRP3 inflammasome was reversible. The LPS‐primed BMDMs were exposed to DCL, followed by three washes to remove any unbound molecules before ATP stimulation. The results showed that the production of IL‐1β remained to be hindered, suggesting the interaction between DCL and NLRP3 was irreversible (Figure 5A). Extensive studies have shown that α,β‐unsaturated lactones can covalently react with the cysteine residues in target proteins through Michael addition. DCL happens to contain such an α,β‐unsaturated lactone that can act as a Michael acceptor to interact with NLRP3 through a covalent bond. To test whether the α,β‐unsaturated lactone in DCL served as the Michael acceptor, we reduced the unsaturated bond to obtain DCL‐R (Figure 5B). Strikingly, DCL‐R did not suppress IL‐1β secretion in BMDMs even at the concentration of 1000 nM (Figure 5C). These results suggest that the α,β‐unsaturated carbonyl moiety is the key pharmacophore in its inactivation of NLRP3 inflammasome.

To elucidate the position of modified cysteine in NLRP3, LC‒MS/MS analysis of digested NLRP3 protein after incubation with DCL was performed to investigate the covalent bond formation. As shown in Figure 5D, the Michael adduct peptide SLGDLIMSCCPDPNPPIHK was identified through a singly charged ion with a molecular mass at 1347.677. The secondary MS of the ion displayed that DCL was bound to Cys280 of NLRP3, in which the m/z of the b10 from the adduct SLGDLIMSCC(DCL)PDPNPPIHK minus that from the control is 230.131, equaling to the molecular weight of DCL. The m/z of the y10 ion further confirmed that the Michael addition occurred on Cys280 of NLRP3. This was corroborated by site‐directed mutagenesis, which showed that mutation of Cys280 (C280A) abolished the binding of DCL to NLRP3 (Figure 5E,F). Additionally, DCL (100 nM) exhibited minimal inhibitory activity on pro‐caspase‐1 and pro‐IL‐1β cleavage in BMDMs expressing the C280A mutant NLRP3 (Figure 5G). Consistently, DCL (3, 10, 30, and 100 nM) exerted little effect on ATPase activity of NLRP3 (Figure 5H). Taken together, these results indicate that DCL can covalently bind to NLRP3, inhibiting its interaction with NEK7 and thereby blocking the activation of NLRP3 inflammasome.

### 2.6 DCL Targets NLRP3 in Macrophages to Alleviate Ulcerative Colitis

**FIGURE 6:**
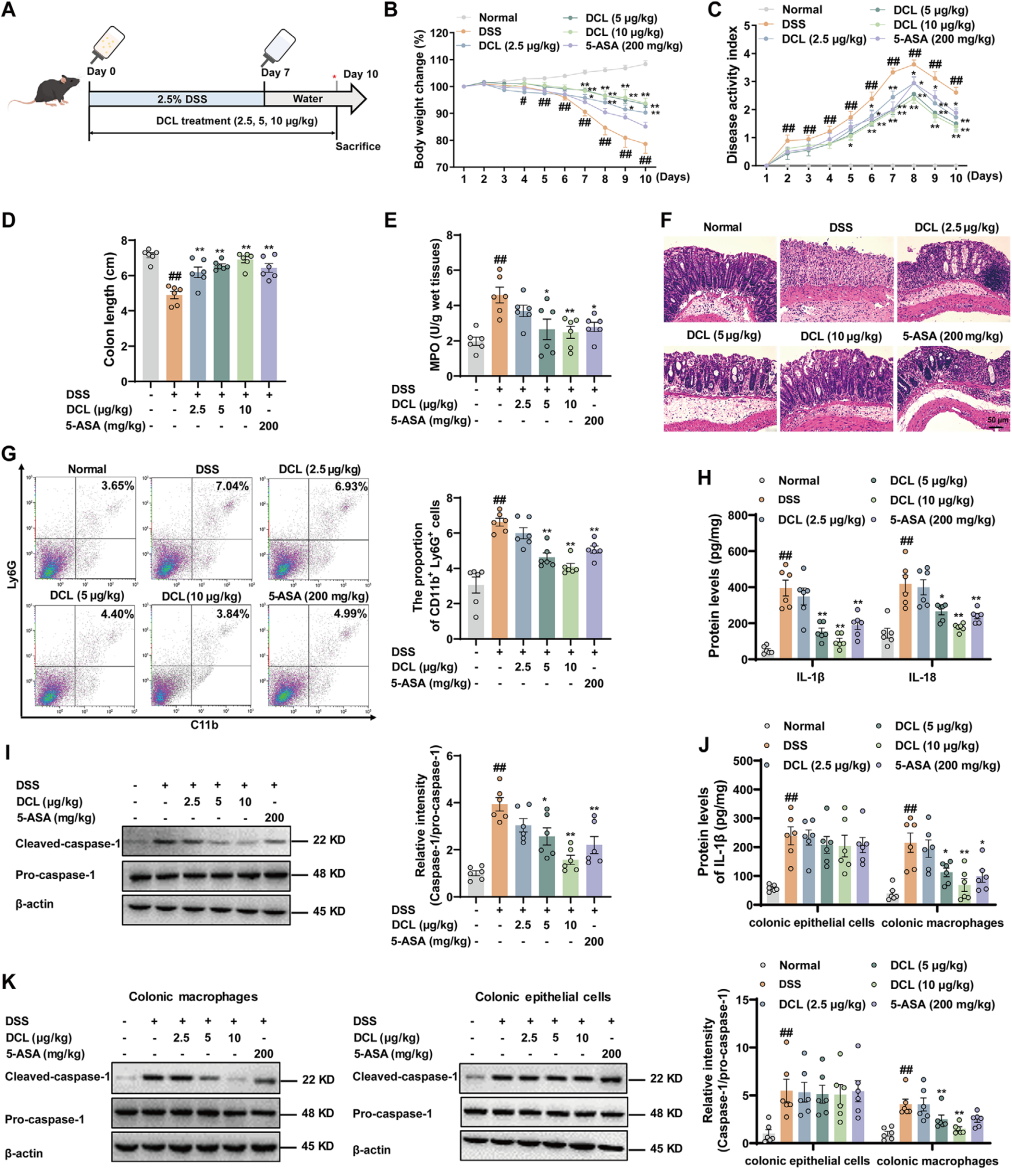
DCL targets NLRP3 in macrophages to alleviate colitis. (A) C57BL/6 mice received 2.5% DSS for the induction of colitis. 5‐ASA (200 mg/kg) and DCL (2.5, 5, and 10 µg/kg) were given to the mice throughout the experiment. (B) Body weight loss. (C) Disease activity index (DAI). (D) Colon length. (E) MPO activity. (F) Hematoxylin and eosin‐stained slides from the colons in mice. (G) The infiltration of neutrophils in colonic lamina propria. (H and J) ELISA analysis of IL‐1β and IL‐18 in colons, colonic macrophages and epithelial cells of mice. (I and K) Western blotting analysis of cleaved caspase‐1 in colons, colonic macrophages, and epithelial cells of mice. Data were presentative of at least of six mice in each group and shown as means ± S.E.M. ^#^ *p* < 0.05 and ^##^ *p* < 0.01 versus normal group. ^*^ *p* < 0.05 and ^**^ *p* < 0.01 versus DSS group.

Previous studies have demonstrated that activation of NLRP3 inflammasome plays a critical role in the pathogenesis of ulcerative colitis (UC). We therefore evaluate the prophylactic efficacy of DCL in a dextran sulfate sodium (DSS)‐induced mouse model of UC (Figure 6A). Here, 5‐aminosalicylic acid (5‐ASA, also known as mesalamine) was listed as the positive control. It is a first‐line anti‐inflammatory drug widely used for the treatment of inflammatory bowel diseases, including UC and Crohn's disease. Its therapeutic effects are primarily attributed to its ability to inhibit cyclooxygenase and lipoxygenase pathways, thereby reducing the production of pro‐inflammatory prostaglandins and leukotrienes. As shown in Figure 6B,C, DCL (5 and 10 µg/kg) dose dependently attenuated clinical symptoms of colitis, including improvements in body weight loss, diarrhea, and rectal bleeding. Additionally, colon shortening and MPO activity in colons were significantly improved by DCL at the indicated dosages (Figure 6D,E). Histological analysis of colons revealed reduced goblet and epithelial cell loss, limited infiltration of inflammatory cells in lamina propria and more complete crypt architecture in DCL‐treated mice (Figure 6F). Flow cytometry analysis showed that infiltration of neutrophils in colonic lamina propria was significantly decreased by DCL (Figure 6G). To further address whether DCL can ameliorate established colitis, DCL was injected intraperitoneally on the fifth day when most mice subjected to DSS developed colitis symptoms (Figure S6A). DCL (5 and 10 µg/kg) markedly reduced disease activity index (DAI) scores, restored colon length, attenuated histopathological changes, inhibited neutrophils infiltration, and decreased MPO activity in colons (Figure S6B‒G). These data indicate that DCL possesses both preventive and therapeutic benefits for DSS‐induced colitis.

Subsequently, the activation of NLRP3 inflammasome in colons of colitis mice was assessed. DCL (5 and 10 µg/kg) markedly inhibited DSS‐induced increase of ASC puncta formation (Figure S7), IL‐1β and IL‐18 secretion in colons of colitis mice (Figure 6H). Furthermore, DCL (5 and 10 µg/kg) notably suppressed the protein expression of cleaved caspase‐1 in colons of DSS‐challenged mice (Figure 6I). Importantly, we observed that DCL specifically restrained the cleavage of caspase‐1 and release of IL‐1β in macrophages rather than epithelial cells isolated from colons [19] (Figure 6J,K). This phenomenon is likely due to the severe loss of colonic epithelial cells in the mouse models, which may have limited the potential effects of DCL on this cell population. To more directly investigate whether DCL alleviated colitis via targeting NLRP3 expressed in macrophages, the F4/80 promoter carrying‐adeno‐associated virus 9 (AAV9) targeting NLRP3 over‐expression was rectal injection into the mice. As expected, DCL‐mediated alleviation of body weight loss, decrease of DAI scores, inhibition of colon shortening, reduction of MPO activity in colons, and improvement of pathological damage in colons was markedly antagonized by AAV9‒NLRP3 (Figure S8A‒E). These findings suggest that DCL restricts NLRP3 inflammasome activation to attenuate colonic inflammation in mice.

### 2.7 DCL Relieves Crohn's Disease in Mice at Extremely Low Dosage

**FIGURE 7:**
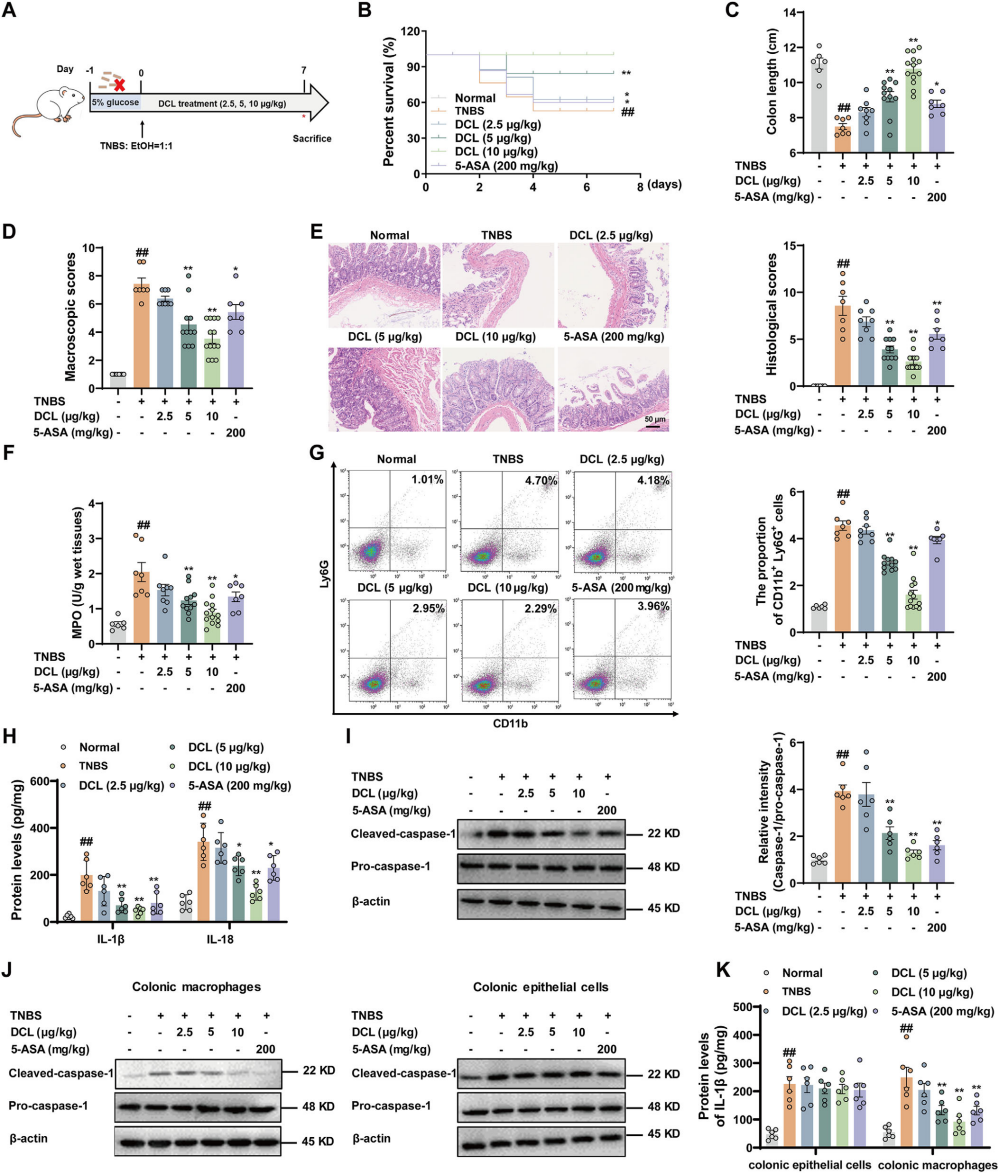
DCL halts NLRP3 inflammasome activation in macrophages to ameliorate Crohn's disease. (A) BALB/c mice were rectally injected with  .5% TNBS to establish colitis model. 5‐ASA (200 mg/kg) and DCL (2.5, 5, and 10 µg/kg) were given to the mice throughout the experiment. (B) The survival proportion of mice. (C) Colon length. (D) The macroscopic score. (E) Hematoxylin and eosin‐stained slides of colons in mice. (F) MPO activity. (G) The infiltration of neutrophils in colonic lamina propria. (H and K) ELISA analysis of IL‐1β and IL‐18 in colons, colonic macrophages, and epithelial cells of mice. (I and J) Western blotting analysis of cleaved caspase‐1 in colons, colonic macrophages, and epithelial cells of mice. Data were presentative of at least of six mice in each group and shown as means ± S.E.M. ^#^ *p* < 0.05 and ^##^ *p* < 0.01 versus normal group. ^*^ *p* < 0.05 and ^**^ *p* < 0.01 versus TNBS group.

Next, we explored the potential preventive efficacy of DCL on 2,4,6‐trinitrobenzenesulfonic acid (TNBS)‐induced Crohn's disease (Figure 7A). DCL‐treated mice exhibited reduced susceptibility to lethal Crohn's disease induction, resulting in a longer survival compared to TNBS‐treated mice (Figure 7B). Furthermore, DCL significantly inhibited colon shortening, reduced colon macroscopic scores, and improved the pathological injury of the colons (Figure 7C‒E). Finally, DCL (5 and 10 µg/kg) dramatically decreased MPO activity in colons and diminished neutrophils infiltration in colonic lamina propria (Figure 7F,G). Collectively, these data indicate a protective role of DCL in TNBS‐induced Crohn's disease.

The activation of NLRP3 inflammasome in colons of mice was also assessed. Similarly, DCL (5 and 10 µg/kg) obviously decreased the ASC puncta formation in colonic tissues (Figure S9). The Western blotting analysis also demonstrated that DCL markedly restrained the cleavage of caspase‐1 and release of IL‐1β in macrophages rather than epithelial cells isolated from colons (Figure 7H‒K). These results demonstrate that inactivation of NLRP3 inflammasome in macrophages is essential for DCL‐mediated improvement of Crohn's disease.

### 2.8 DCL Mitigates LPS‐Induced Septic Shock and MSU‐Induced Peritoneal Inflammation

**FIGURE 8:**
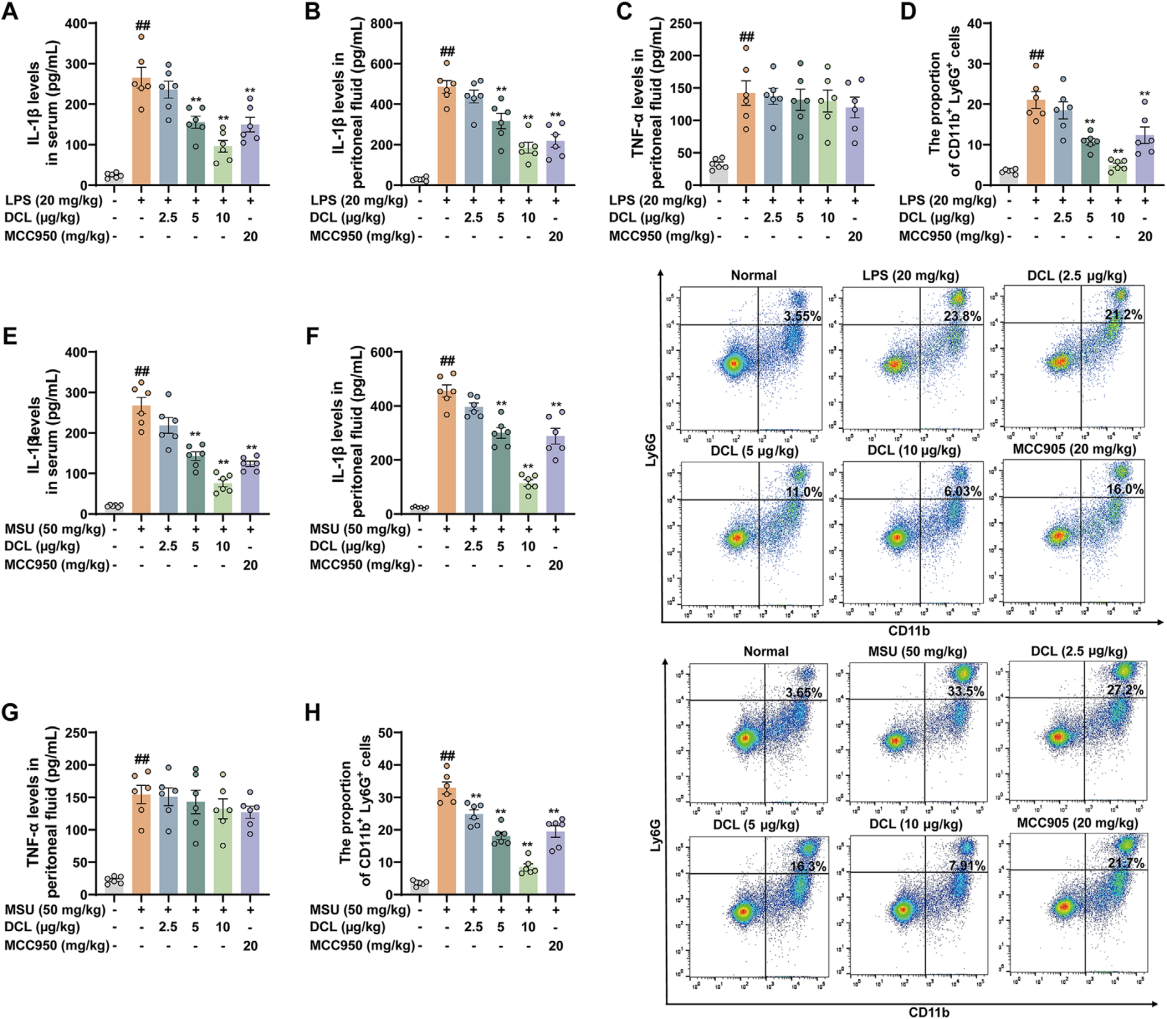
DCL mitigates LPS‐induced septic stock and MSU‐induced peritonitis through inhibiting NLRP3 inflammation activation. The mice were intraperitoneally injected with DCL (2.5, 5, and 10 µg/kg), 1 h later, the mice were further intraperitoneally injected with LPS (20 mg/kg) to induced septic shock or MSU (50 mg/kg) to induced peritonitis. Four hours later, the mice were euthanized for the following analysis. (A‒C and E‒G) ELISA analysis of IL‐1β and TNF‐α secretion in the peritoneal lavage fluids and serum. (D and H) Flow cytometry analysis of the neutrophils in peritoneal lavage fluids. Data were presentative of at least of six mice in each group and shown as means ± S.E.M. ^#^ *p* < 0.05 and ^##^ *p* < 0.01 versus normal group. ^*^ *p* < 0.05 and ^**^ *p* < 0.01 versus LPS or MSU group.

To assess the protective effect of DCL against LPS‐induced septic shock mediated by NLRP3 inflammasome activation, the mice were pre‐treated with DCL prior to intraperitoneal injection of LPS. Subsequent measurement of pro‐inflammatory cytokines levels showed that DCL (5 and 10 µg/kg) effectively inhibited IL‐1β but not TNF‐α production in peritoneal lavage fluid and serum (Figure 8A‒C). The flow cytometry analysis showed that DCL reduced the proportion of neutrophils in peritoneal lavage fluid (Figure 8D). Furthermore, the protective effect of DCL against MSU‐induced peritonitis mediated by NLRP3 inflammasome activation was investigated. Consistently, DCL (5 and 10 µg/kg) significantly attenuated MSU‐induced IL‐1β production and reduced the percentage of neutrophils in peritoneal lavage fluid and serum (Figure 8E‒H). Collectively, these results underscore the efficacy of DCL in preventing peritonitis through inhibition of NLRP3 inflammation activation.

Having identified the protective efficacy of DCL against UC, Crohn's disease, septic shock, and peritonitis, we then investigated its safety profile in healthy mice. Importantly, continuous administration of DCL (2.5, 5, and 10 µg/kg) for 10 days did not influence the body weight, colon length, main organ weight, or their histological appearance (Figure S10A‒D). DCL (2.5, 5, and 10 µg/kg) exerted minimal effect on the percentage of CD3^+^ CD8^+^ T cells, CD3^+^ CD4^+^ T cells, F4/80^+^ CD11b^+^ macrophages, CD19^+^ B cells, CD11b^+^ Ly6G^+^ neutrophils, and CD11b^+^ Ly6C^+^ monocyte (Figure S10E). Additionally, the serum levels of aspartate aminotransferase, alanine aminotransferase, creatinine, and urea nitrogen remained unchanged following DCL treatment, suggesting that DCL in current solution and dosing is safe (Figure S10F‒I). More importantly, even at higher dosages of 30 and 100 mg/kg, DCL had negligible effects on the healthy mice (Figure S11A‒I). These findings sum up that DCL is a potent and safe therapeutic candidate for the treatment of NLRP3‐associated inflammatory diseases.

## 3 Discussion

DCL is the main active component of *Aucklandiae lappa* Decne, a traditional Chine medicine primarily used to treat digestive system diseases including 5‐fluorouracil‐induced intestinal mucositis, irritable bowel syndrome, etc. [20]. In the present study, we demonstrated that DCL effectively impaired the cleavage of pro‐caspase‐1 and release of IL‐1β and IL‐18 at the concentration of 10 nM in both mouse and human macrophages. Mechanistically, DCL covalently binds to Cys280 of NLRP3, exhibiting an extremely potent inhibitory effect on NLRP3 inflammasome activation. Notably, DCL exhibited significantly superior inhibitory potency than oridonin, with an IC_50_ value of 25.44 nM versus 274.67 nM. Simultaneously, DCL significantly ameliorated colonic inflammation, septic shock, and peritonitis in mice at the dosage of 5 µg/kg. More importantly, even at higher dosages of 30 and 100 mg/kg, DCL showed negligible toxicity in healthy mice, underscoring its favorable safety profile and strong potential as a candidate for NLRP3‐associated inflammatory diseases.

Previously reports have indicated that DCL possesses the ability to block the activation of MAPK and NF‐κB pathways [21, 22], the first signal for NLRP3 inflammasome activation, thereby reducing the generation of IL‐6 and TNF‐α. However, we found that the concentration needed for inactivation of MAPK and NF‐κB signaling pathways was approximately 1000 times higher than that required for NLRP3 inflammasome inhibition (30 nM vs. 30 µM), suggesting that DCL exerted its anti‐inflammatory effects primarily by suppressing NLRP3 inflammasome activation. Additionally, Chen et al. documented that DCL halted NLRP3 inflammasome activation through inhibiting ASC polymerization [23], but the definitive target was still unknown. Our results propose that DCL directly covalently binds to NLRP3, thereby inhibiting NLRP3 inflammasome assembly and ultimately exerting anti‐inflammatory effects.

It is well known that the components of NLRP3 inflammasome, upstream signaling events, and effectors including caspase‐1 and IL‐1β can be targeted for therapeutic strategies against NLRP3‐related inflammatory diseases. Drugs targeting IL‐1β include the IL‐1R monoclonal antibody anakinra [24], IL‐1β neutralizing antibody canakinumab [25], and rilonacept [26]. However, several disadvantages of these biologics cannot be ignored: (i) NLRP3 inflammasome activation produces not only IL‐1β but also IL‐18, which may be involved in pathogenic and immunomodulatory process [27]; (ii) most biologics cannot be administered orally and have limited ability to penetrate the blood‒brain barrier; (iii) IL‐1β produced by other inflammasomes may have normal physiological functions, and blockage of IL‐1β increases the risk of opportunistic infections [28]. Other agents disturbing upstream signaling events of NLRP3 inflammasome activation including β‐hydroxybutyrate (BHB), cardamonin, sulforaphane, etc. Sulforaphane reduces ROS production via enhancing AMPK‐induced autophagy pathway, thereby inhibiting NLPR3 inflammasome activation [29]. However, sulforaphane does not selectively target NLRP3 inflammasome [30]; cardamonin inhibits NLPR3 inflammasome activation by agitating aryl hydrocarbon receptor [31], but it can lead to adverse reactions including liver injury and intestinal obstruction [32]; BHB blocks NLRP3 inflammasome activation through decreasing K^+^ efflux [8], but it also halts NF‐κB signaling transduction [33]. In summary, they are not ideal candidates for the therapy of NLRP3 inflammasome‐related inflammatory diseases due to the non‐specific actions and potential adverse effects.

Targeting NLRP3 itself can specifically block its activation and mediate superior anti‐inflammatory effects. Previous studies have shown that some small molecule inhibitors can directly target NLRP3, thereby inhibiting NLRP3 inflammasome activation by hindering its ATPase activity. For instance, MCC950 blocks NLRP3 inflammasome activation at nanomolar concentrations (IC_50_ = 10 nM) by binding to the walker B motif of NLRP3's NACHT domain, preventing ATP hydrolysis and NLRP3 inflammasome assembly [34]. However, MCC950 was terminated in phase II clinical trials due to serious hepatotoxicity [35]. CY‐09, another inhibitor, directly binds to the walker A motif of NLRP3's NACHT domain and inhibits its ATPase activity and multimerization, thereby specifically blocking NLRP3 inflammasome assembly and activation. Although CY‐09 can relieve inflammation and insulin resistance of patients suffered from T2D, it greatly causes body weight loss in patients [36]. Therefore, the discovery and development of novel NLRP3 inflammasome inhibitors with potent activity and high safety have important research and clinical values.

In the present study, DCL did not affect the ATPase activity of NLRP3. More importantly, DCL was the first natural compound to covalently and irreversibly interact with Cys280 of NLRP3, a mechanism completely different from MCC905. In addition, DCL did not affect upstream signals including K^+^ efflux, Ca^2+^ influx, Cl^−^ efflux, or mitochondrial ROS generation. We showed that DCL bound to NLRP3 itself to block NLRP3 inflammasome activation primarily by reducing the interaction between NEK7 and NLRP3, a critical step in inflammasome assembly. Since the full length NLRP3 protein is composed of 1036aa and contains 45 cysteine residues, further exploration is needed to explored why DCL specifically binds to Cys280 of NLRP3 and how this interaction inhibits NEK7 binding to NLRP3.

Based on the above considerations, pharmacists have developed various covalently bound drugs, including as aspirin, penicillin, Fluorouracil, Fosfomycin, Omeprazol, Clopidogrel and Bortezomib, Boceprevir, Cafilzomib, Afatinib, Osimertinib, Voxelotor, and Sotorasib, which have significant impact on human health [37]. The covalently bonded drugs have a series of pharmacological advantages: (i) they can achieve full target occupancy even at relatively low concentrations, thereby enhancing biochemical efficiency; (ii) they can prolong the duration of action, thereby reducing the frequency of administration [38]; (iii) they are less sensitive to pharmacokinetic parameters and can maintain efficacy even if the drug is rapidly cleared from the body [39]; (iv) as long as the covalent binding pattern is not affected by mutations, they are generally less susceptible to drug resistance caused by chemotherapy mutations [40]; (v) the covalent warheads can target rare, non‐conserved amino acid residues of particular proteins, thereby achieving high selectivity and reducing off‐target reactions. Based on the above‐mentioned advantages, we first reported that DCL can directly bind to NLRP3 through a covalent bond. More importantly, DCL has a highly potent anti‐colitis, Crohn's disease, septic shock, and peritonitis effects in vivo. Therefore, DCL and its analogues may serve as promising therapeutic agents for the treatment of NLRP3‐mediated inflammatory diseases.

In summary, through the virtual screening of diverse natural product libraries, DCL was identified as a potential NLRP3 inhibitor. Detailed experiments verified that DCL possessed extremely potent and selective NLRP3 inhibitory activity. Furthermore, DCL significantly ameliorated colitis, Crohn's disease, septic shock, and peritonitis in mice at an extremely low dosage of 5 µg/kg. Deeper mechanistic studies revealed that the α,β‐unsaturated carbonyls structure of DCL served as a pharmacophore, forming an irreversible covalent bond with Cys280 of NLRP3. This covalent interaction significantly inhibited the formation of NEK7/NLRP3 complex and subsequent assembly of NLRP3 inflammasomes. More importantly, this novel binding mode ensured that DCL did not affect the ATPase activity of NLRP3. Therefore, our study provides a promising candidate for clinical treatment of NLRP3‐related diseases.

## 4 Materials and Methods

### 4.1 Isolation of BMDMs

The mice were anesthetized by isoflurane, killed by dislocation of cervical vertebra and sterilized in 75% alcohol. Subsequently, the femur on both sides was cut off, and the attached muscle was peeled. The bone marrow was gently flushed out into a 15 mL centrifuge tube with RPMI 1640 medium and then subjected to centrifugation at 1700 rpm for 7 min. After that, the pellets were re‐suspended in complete RPMI 1640 medium and cultured in 10 cm dish for 24 h. The no adherent cells were cautiously gathered and cultured in RPMI 1640 medium containing 20% conditioned medium from L929 cells for 3 days. Subsequently, half old medium was replaced with fresh complete media (containing 20% conditional medium from L929 cells). On day 7, the BMDMs were ready for subsequent experiments.

### 4.2 Inflammasome Activation

BMDMs and HMDMs were plated in six‐well cell plates and then primed with 100 ng/mL ultrapure LPS (Sigma, L6529, Saint Louis, MO, USA) for 4 h. After that, the cells were cultured with DCL (10, 30, and 100 nM) for 1 h, followed by stimulation with NLRP3 inflammasome agonists including 5 mM ATP (Sigma, A7699, Saint Louis, MO, USA, 45 min), 4 µM nigericin (Invivogen, tlrl‐nig, San Diego, CA, USA, 1 h), and 150 µg/mL MSU (Invivogen, tlrl‐msu, San Diego, CA, USA, 6 h). Activation of AIM2, NLRC4, and NLRP1 inflammasome in LPS‐primed cells was achieved by cultured with 1.5 µg poly(dA:dT) (Invivogen, tlrl‐patn, San Diego, CA, USA) for 12 h, 200 ng/mL purified flagellin from *Pseudomonas aeruginosa* (Invivogen, tlrl‐pafla, San Diego, CA, USA) for 2.5 h, and 200 ng/mL MDP (Invivogen, tlrl‐mdp, San Diego, CA, USA) for 6 h.

### 4.3 ASC Oligomerization

The NLRP3 inflammasome activation model was established in BMDMs as mentioned above. Then, the cells were scraped with cell scraper and lysed using NP40 lysis buffer containing protease inhibitor cocktail for 30 min on ice. The samples were centrifuged for 10 min at 6000 rpm, and the cell pellets were cross‐linked with 500 µL of PBS containing 2 mM disuccinimidyl sulphate (Sangon Biotech, DSS, C100015, Shanghai, China) for 35 min at 37°C. After centrifugation for 15 min at 330 g, the cross‐linked protein lysates were re‐suspended with 30 µL 2× SDS loading buffer (Beyotime Biotechnology, P0015B, Shanghai, China). After boiling for 10 min at 100°C, the proteins were used for Western blotting immediately or stored in a ‒80°C freezer.

### 4.4 ASC Speck Formation

The NLRP3 inflammasome activation model was established in BMDMs seeded in confocal dish as described above. Subsequently, 0.5 mL of 4% paraformaldehyde was added into each well for 20 min to fix the cells and avoid clumping. Subsequently, the cells were permeabilized with 0.1% Triton X‐100 (Beyotime Biotechnology, ST795, Shanghai, China), blocked with 3% BSA and cultured with anti‐ASC antibody (Cell Signaling Technology, 67824, RRID: AB_2799736, Danvers, MA, USA) overnight at 4°C. The next day, Alexa Fluor 488‐conjugated goat anti‐rabbit IgG (Invitrogen, A11034, RRID: AB_2576217, Waltham, MA, USA) secondary antibody was added to the cells and the cocktail was incubated for 1 h at 37°C. Lastly, the cells were dyed with DAPI (Servicebio, G1012, Wuhan, China) for 8 min in dark, and the cells were visualized under Leica TCS SP8 X (Leica, Germany) confocal microscope using a 63× objective.

### 4.5 Co‐immunoprecipitation

For the endogenous interaction assay, the BMDMs or HMDMs were primed with LPS (100 ng/mL) for 4 h, followed by treatment with DCL (3, 10, 30, and 100 nM) for 1 h. Cells were then stimulated with ATP (5 mM) for 45 min to active the NLRP3 inflammasome. Subsequently, the cells were lysed in ice‐cold lysis buffer (P0013, Beyotime Biotechnology, Shanghai, China) for 30 min on ice, followed by centrifugation at 12,000 × *g* for 15 min at 4°C to remove cell debris. The supernatant was incubated with anti‐ASC (67824, 1:100, Cell Signaling Technology, Inc., Danvers, MA, USA) or anti‐NLRP3 (68102‐1‐Ig, 1:100, Proteintech, Wuhan, China) primary antibody or control IgG at 4°C overnight with gentle rotation. Protein A/G agarose beads (BD0045, Biogot Technology Co., Ltd., Nanjing, China) were then added and incubated for an additional 4 h at room temperature. The immune complexes were washed three times with lysis buffer and eluted by boiling in SDS loading buffer. The samples were then subjected to SDS‐PAGE and analyzed by Western blotting using the NEK7 or pro‐caspase‐1 primary antibodies. For the exogenous interaction assay, the HEK‐293T cells were transfected with plasmids (HA‐NEK7, His‐NLRP3) in six‐well plates through the Lipofectamine 3000 (Thermo Fisher Scientific, A11034, L3000015, Waltham, MA, USA). After 24 h, the cells were collected and lysed with NP‐40 lysis buffer with complete protease inhibitor. Extracts were immunoprecipitated with anti‐His antibody and the protein A/G agarose beads were then assessed by Western blotting analysis.

### 4.6 Pull‐Down Assay

For the pull‐down assay, biotinylated DCL‐P was incubated with lysates prepared from BMDMs transfected with NLRP3 or NEK7 plasmids. Cell lysates were prepared using NP‐40 lysis buffer and incubated with streptavidin‐conjugated magnetic beads at 4°C for 4 h with gentle rotation. After incubation, the beads were washed five times with lysis buffer to remove nonspecific binding. The bound proteins were then eluted by boiling in SDS loading buffer and analyzed via Western blotting to detect interactions between DCL‐P and NLRP3 or NEK7.

### 4.7 Animal

Six to 8 weeks old female C57BL/6 mice and male BALB/c mice were provided by Shanghai Slack Laboratory Animal Co., Ltd. (Shanghai, China). All animals were confirmed to be healthy and had no prior exposure to experimental procedures. The mice were housed in individually ventilated cages under specific pathogen‐free conditions, maintained at a temperature of 20°C‒26°C and relative humidity of 30%–70%, with a 12‐h light/dark cycle. Animals were provided with ad libitum access to autoclaved food and water. Prior to the experimentation, mice were acclimatized for at least 7 days. All experimental procedures and animal care have been inspected and approved by the animal ethics committee of Nanjing University of Chinese Medicine (approved numbers: 202207A027, 202209A035, and 202503A062).

### 4.8 DSS‐Induced Ulcerative Colitis

For the assessment of protective effect of DCL against colitis, the mice were randomly allocated into normal, DSS, 5‐ASA (200 mg/kg), and DCL (2.5, 5, and 10 µg/kg) groups based on body weight. Except for the normal group, the rest mice were provided with 2.5% DSS in the drinking water for 7 days, and subsequently switched to normal water for 3 days to induce colitis. DCL and 5‐ASA were dissolved by olive oil and 0.5% sodium carboxy methyl cellulose, respectively. Subsequently, the DCL was administered to mice via intraperitoneal injection and 5‐ASA was given by gavage daily. They were sacrificed at the endpoint of treatment schedule. The colons were collected, and their length from the cecum to the anus was measured with a centimeter ruler.

### 4.9 TNBS‐Induced Crohn's Disease

All BALB/c mice were adaptively fed for 7 days before the establishment of this model, and then they were fasted for 24 h with free access to 5% glucose solution during this period. They were randomly allocated into normal, TNBS, 5‐ASA (200 mg/kg), and DCL (2.5, 5, and 10 µg/kg) groups based on the body weight. To induce colitis, 100 µL of ethanol‐diluted 1.5% TNBS (Sigma, P2297, Saint Louis, MO, USA) was slowly instilled into colon of the mice by gavage needle. After the TNBS solution is injected, mice were maintained in a head‐down position for 3 min to prevent TNBS or ethanol from leaking. At the same day, 5‐ASA and DCL were given for 7 consecutive days according to the aforementioned method.

### 4.10 LPS‐Induced Systemic Inflammation

The C57BL/6 mice were randomly divided into five groups: normal, LPS, and DCL (2.5, 5, and 10 µg/kg) groups based on the body weight. They were intraperitoneally administered with DCL (2.5, 5, and 10 µg/kg) for 1 h, followed by an i.p. injection of LPS (10 mg/kg). Four hours post‐injection, the mice were euthanized, and the serum samples were harvested. The peritoneal cavities were rinsed with 10 mL of ice‐cold PBS. The levels of plasma IL‐1β was quantified using ELISA, the polymorphonuclear neutrophils in peritoneal lavage fluid were identified and quantified by flow cytometry after staining with Ly6G and CD11b.

### 4.11 MSU‐Induced Peritonitis

The C57BL/6 mice were randomly divided into five groups: normal, MSU, and DCL (2.5, 5, and 10 µg/kg) groups based on the body weight. They were i.p. administered with DCL (2.5, 5, and 10 µg/kg) for 1 h, followed by an i.p. injection of MSU crystals (50 mg/kg). Six hours post‐injection, the mice were euthanized by cervical dislocation. The blood was collected, and serum samples were isolated. The peritoneal cavity was rinsed with 10 mL of ice‐cold 1× PBS, and the peritoneal lavage fluid was harvested. Levels of IL‐1β in both the serum and peritoneal lavage fluid were assessed using ELISA. The peritoneal exudative cells and the number of polymorphonuclear neutrophils in peritoneal lavage fluid were determined by flow cytometry.

### 4.12 Statistics

Graph Prism 8.0 (GraphPad Software, San Diego, CA, USA) was used for statistical analysis of data, and the measurement data was expressed in mean ± standard error of mean. The homogeneity of variance among multiple groups compared by one‐way analysis of variance. The difference was statistically significant with *p* < 0.05.

## Author Contributions

Lihong Hu and Jian Liu conceived of the study and designed the experiments. Qi Lv, Juan Wang, and Weijiang Lin interpreted the experiments, prepared the images for publication, and drafted the manuscript. Yishu Zhang carried out the new experiments and organized all the original Western blotting strips. Ying Xie, Hongqiong Yang, Xunkai Yin, Zhenzhen Zhu, and Yifan Cui performed the in vitro and in vivo experiments and collected the data. Yang Hu provided DCL in the study. Li Zeng and Yinan Zhang aided in experimental design, provided technical support, and proofread the manuscript. Xubing Chen provided the dehydrocostus lactone and proofread the manuscript. All the authors have read and approved the final manuscript.

## Ethics Statement

The healthy volunteers in the present study provided their informed consent, and the protocol was approved by Ethics Committee of The Second Hospital of Nanjing (approved number: 2022‐LS‐ky017). All animal experiments in the present study were conducted in accordance with the Guide for the Care and Use of Laboratory Animals, and the protocol was approved by the Animal Ethics Committee of Nanjing University of Chinese Medicine (approved numbers: 202207A027, 202209A035, and 202503A062).

## Conflicts of Interest

All authors declare no conflicts of interest.

## Supporting information




**Supporting File**: mco270367‐sup‐0001‐SuppMat.pdf

## Data Availability

Data supporting the present study are available from the corresponding author upon reasonable request.
