# Supplementary material for: Dehydrocostus Lactone Effectively Alleviates Inflammatory Diseases by Covalently and Irreversibly Targeting NLRP3
Source: MedComm (2020). 2025 Sep 3;6(9):e70367. doi: 10.1002/mco2.70367 (PMC12408923; doi:10.1002/mco2.70367)
Supplement: Supplementary file 1 — Supporting File: mco270367‐sup‐0001‐SuppMat.pdf [file MCO2-6-e70367-s001.pdf]

Dehydrocostus lactone effectively alleviates inflammatory diseases by covalently and irreversibly targeting NLRP3

1. Material and Methods

1.1 Cell culture

L929 (RRID: CVCL_0462) cells were purchased from the American Type Culture Collection (ATCC) and authenticated by short tandem repeat (STR) analysis. The cells were grown in RPMI1640 medium supplemented with 10% fetal bovine serum (FBS, Biological Industries Ltd., Israel), 1% penicillin/streptomycin (P1400, Solarbio Life Science, Beijing, China). Cultures were maintained at 37 °C in a 5% CO_2 incubator.

1.2 Conditional medium from L929 cells

L929 cells after rapid recovery were seeded on the 10 cm dishes at 37 °C in a 5% CO_2 incubator. When the cell confluence reached 70%-80%, 12 mL of RPMI 1640 medium containing 10% FBS was added. 5-6 days later, the supernatant was collected into a 15 mL centrifuge tube, stored in -20 °C freezer, and filtered with 0.22 μm filter when used.

1.3 Differentiation of human peripheral blood monocytes (PBMCs)

The PBMCs were obtained from whole umbilical cord blood collected from healthy, microbiologically screened volunteers by centrifugation on a density gradient. All research participants have signed their informed consent, and all the procedures were approved by the Institutional Research Ethics Committee of The Second Hospital of Nanjing (approved number: 2022-LS-ky017). The fresh anticoagulant whole blood was taken and diluted with equal volume PBS. Then, the proper volume of separation solution (Solarbio Life Science, P8610, Beijing, China) was added on to the 15 mL centrifuge tube, followed by addition of diluted fresh blood on the surface. After centrifugation at 1600 rpm for 30 min on horizontal rotor, there will be obvious stratification: plasma layer, separation liquid layer, white membrane between plasma

and separation liquid. The white membrane layer was carefully extracted and the cells were obtained as PBMCs. To further obtain HMDMs, the PBMCs were cultured for 7 days in a humidified 37 °C, 5% CO_2 with RPMI 1640 medium containing 20 ng/ml of recombinant human macrophage colony-stimulating factor (rhM-CSF, Peprotech Inc., 300-25, Waltham, MA, USA).

1.4 Real-time polymerase chain reaction

Total RNA was extracted by Trizol reagent (R4010-01, Vazyme, Nanjing, China) following the manufacturer's instructions strictly. The concentration of the extracted RNA was quantified in NanoDrop spectrophotometer (Thermo Fischer, Waltham, MA, USA), and the purity was determined by the ration of absorbance wavelength at 260 and 280 nm. A ratio close to 2.0 is generally considered indicative of "pure" RNA suitable for further experiment. Subsequently, a total of 0.5 μg RNA was reverse-transcribed to cDNA using HiScript Q RT SuperMix (R312-01, Vazyme, Nanjing, China). Quantitative PCR reactions were conducted with cDNA, SYBR Green Master Mix (11201ES03, Shanghai, China), and 1 μM forward and reverse primes using LightCycler® 96 PCR system (Roche, Basel, Switzerland). The relative mRNA levels of target genes were quantified by $2^{-\Delta\Delta Ct}$ method based on the amplification results. The primer sequences applied in this experiment were all shown in Table S1.

1.5 Western blotting

To extract the total protein from lysate, the cells were lysed by NP-40 buffer containing a proteinase inhibitor (ST507, Beyotime Biotechnology, Shanghai, China) on ice for 30 min. The cell lysates were then centrifuged at 12000 rpm for 10 min at 4 °C, and the supernatant was gently gathered and mixed with 5× SDS-PAGE loading buffer. For protein extraction from supernatants, 500 μL of cell culture was collected in a 1.5 mL EP tube, and 125 μL of chloroform was added. The mixture was vortexed for 3 min and then centrifuged at 12000 rpm for 10 min at 4 °C. The upper layer solution was discarded, and 500 μL of methanol was added. After vortexing for 5 min, the mixture was centrifuged at 12000 rpm for another 10 min to obtain protein in

supernatant.

An equal amount of protein was loaded to 8% or 10% SDS-PAGE wells and transferred onto PVDF membranes. Then, the membranes were blocked by Tris-buffered saline with 0.05% Tween-20 (TBST) containing 5% nonfat milk for 2 h on decoloring shaker and probed with different primary antibodies of NLRP3 (15101, 1: 1000, CST, Danvers, MA, USA), ASC (67824, 1: 1000, CST), caspase-1 (24232, 1:1000, CST), IL-1β (31202, 1: 1000, CST), cleaved caspase-1 (89332, 1: 1000, CST), cleaved IL-1β (63124, 1: 1000, CST) at 4 °C for overnight. After washing with TBST for three times, membranes were incubated with the indicated horseradish peroxidase (HRP)-linked anti-rabbit (7074, 1: 1000, CST) or anti-mouse (7076, 1:1000, CST) secondary antibodies for 1 h at room temperature. The final detection was performed by SuperSignal™ West Femto (34094, Thermo Fisher Scientific).

1.6 Mitochondrial ROS measurement

The NLRP3 inflammasome activation model was established in BMDMs as described above. The cells were rinsed with PBS, followed by cultivation with with 100 nM Mito-Tracker Red CMXRos (Beyotime Biotechnology, C1049, Shanghai, China) at 37 °C for 20 minutes in the dark. Next, the nucleus was dyed with DAPI (Servicebio, G1012, Wuhan, China) for 8 min. Finally, mitochondrial ROS (mitoROS) generation was visualized under confocal microscopy or analyzed by flow cytometry on Gallios instrument (Beckman Coulter Inc., Pasadena, CA, USA).

1.7 Intracellular K^+ and Ca^{2+} measurement

The NLRP3 inflammasome activation model was established in BMDMs as previously described. Then, the Ca^{2+}-sensitive fluorescent dye Fluo-4 acetoxymethyl ester (Beyotime Biotechnology, S1060, Shanghai, China) or ION Potassium Green-2AM (Abcam, ab142806, Cambridge, UK) was added and cultured in the dark at 37 °C for 25 min. After that, they were gently rinsed with D-Hanks solution three times, and the content of calcium ion as well as potassium ion was analyzed by flow cytometry on Gallios instrument (Beckman Coulter Inc., Pasadena, CA, USA).

1.8 Intracellular Cl^- measurement

For accurate measurement of the intracellular chloride, BMDMs were plated overnight in 12 well plates and then primed with 100 ng/mL LPS for 3 h. After that, cells were treated with DCL for 1 hour and then stimulated with ATP for 30 min. The supernatants of 12 well plates were removed, and ddH_2O was added (200 μl per well) and kept for 15 min at 37 °C. The lysates were transferred to 1.5 mL EP tube and centrifuged at 10, 000 × g for 5 min. 50 μL supernatants was then transferred the to a new 1.5 mL EP tube and mixed with 50 μL macrophage quinacrine analog (MQAE, 10 μM). The fluorescence intensity was measured at Excitation 350 nm and Emission 460 nm using the BioTek Multi-Mode Microplate Readers. A control was settled in every experiment to determine the extracellular amount of chloride remaining after aspiration, and this value was subtracted.

1.9 Surface plasmon resonance (SPR)

The affinity analysis between DCL and rhNLRP3 protein was performed on the Biacore T200 instrument (GE Healthcare, Boston, MA, USA). Firstly, the rhNLRP3 protein (Sinaglway Antibody LLC, GP10109-1, College Park, MD, USA) was immunobilized on the Fc2 channel of CM5 chip (GE Healthcare, BR100012, Boston, MA, USA) by 1-ethyl-3(3-dimethylaminopropyl) carbodiimide and N-hydroxysuccinimide mediated amine coupling reaction. Then, the surface of chip was blocked with 1 M Ethanolamine hydrochloride-NaOH for 420 s. The reference Fc1 channel underwent consistent process but without the binding of rhNLRP3 protein. Different concentrations of DCL samples prepared in a running buffer (1.05 × PBS-P, 5% DMSO, PH 7.4) was run over the Fc2-Fc1 channels on the instrument. The SPR parameters were set as follows: contact time, 120 s; disassociation time, 180 s; flow rate, 30 μL/min; temperature, 25 °C. The final disassociation constant K_D was analyzed by using Biacore T200 software Evaluation Software (GE Healthcare, Boston, MA, USA).

1.10 Immunofluorescence

One milliliter of 4% paraformaldehyde (Beyotime Biotechnology, P0098, Shanghai, China) was added to the BMDMs, colonic tissue slice and incubated at RT for 20 min. The cells and sections were washed with ice-cold PBS three times and then permeabilized with 0.1% Triton X-100 (Beyotime Biotechnology, P0096, Shanghai, China) for 20 min. After three washes with pre-chilled PBS, the cells and sections were blocked with 1% BSA (Beyotime Biotechnology, P0102, Shanghai, China) for 2 h and incubated with NLRP3 (Proteintech, 68102-1-Ig, RRID: AB_2923634, Wuhan, China) or ASC (Cell Signaling Technology, 67824, RRID: AB_2799736, Danvers, MA, USA) overnight at 4 °C. The next day, the cells and sections were stained with Alexa Fluor 488 goat anti-mouse secondary antibody (Servicebio, GB25301, RRID: AB_2904018, Wuhan, China) or Cy5 goat anti-rabbit secondary antibody (Servicebio, GB27303, RRID: AB_2905513, Wuhan, China) in dark for 1 h. After that, the DAPI (Servicebio, G1012, Wuhan, China) was applied to the cells and sections to detect the cell nucleus, and the confocal images were acquired using a Leica TCS SP8 X (Leica, Germany).

1.11 Cellular thermal shift assay

BMDMs were scraped into EP tubes and lysed in a rotating shaker for 20 minutes at 4 °C, followed by centrifugation at 16,000g for 15 minutes. The protein was equally divided into two groups: one group was incubated with DCL and the other group was incubated with DMSO for 1 hour at room temperature (RT). Subsequently, each group was divided into 10 aliquots, and subjected to heating at different temperatures for 3 minutes each. After completion of the heating process, the cocktail was centrifuged for 20 min at 16,000g, and the protein levels of NEK7 and NLRP3 were assessed by western blotting assay.

1.12 Solvent-induced protein precipitation

BMDMs pretreatment process was consistent with the above scheme. Subsequently, the supernatant was divided into 10 equal portions and precipitated with acetone/ethanol/acetic acid (A.E.A=50: 5: 0.1). The final organic solvent percentage reached 9% to 19%, and the mixture was then incubated at 800 rpm on thermomixer

for 30 minutes. The cocktail was further centrifuged for 20 min at 16,000g, the cell supernatants were harvested and the protein levels of NLRP3 or NEK7 were measured by performing western blotting assay.

Table S1. Primers used in qPCR

Primers		Sequence (5’-3’)
Actb (mouse)	Forward	CTACCTCATGAAGATCCTGACC
	Reverse	CACAGCTTCTCTTTGATGTCAC
Il6 (mouse)	Forward	CTCCCAACAGACCTGTCTATAC
	Reverse	CCATTGCACAACTCTTTTCTCA
Tnf (mouse)	Forward	ATGTCTCAGCCTCTTCTCATTC
	Reverse	GCTTGTCACTCGAATTTTGAGA
Nlrp3 (mouse)	Forward	GCCGTCTACGTCTTCTTCCTTTCC
	Reverse	CATCCGCAGCCAGTGAACAGAG

Supplementary Figures

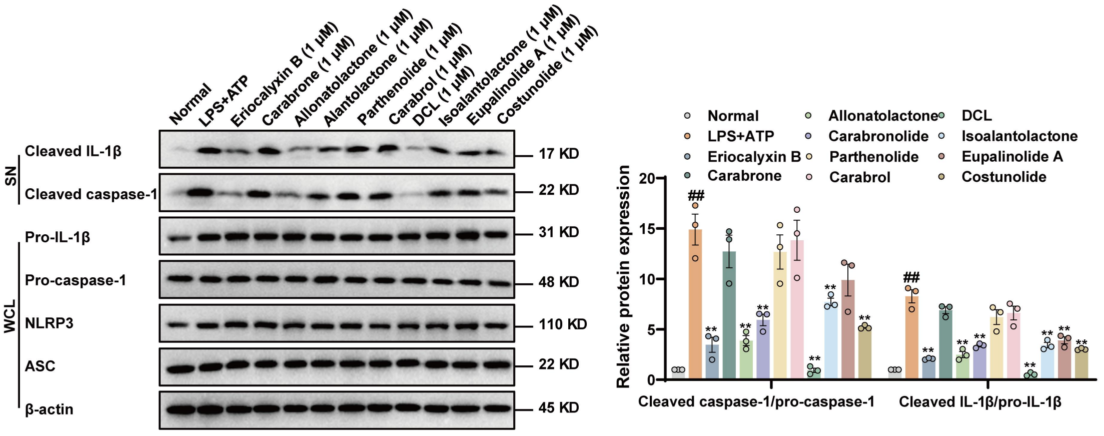

Supplementary Figure 1. The effect of ten potential NLRP3 inhibitors on inflammasome activation in BMDMs. The BMDMs were primed with LPS (100 ng/mL) for 4 hours, followed by incubation with Eriocalyxin B, Carabrone, Allonatolactone, Alantolactone, Parthenolide, Carabrol, DCL, Isoalantolactone, Eupalinolide A or Costunolide at the concentration of 1 μM for 1 h. Subsequently, the cells were treated with ATP (5 mM) for 45 min. the protein expressions of cleaved caspase-1 and cleaved IL-1β in supernatant were detected by western blotting assay. Data from *in vitro* assays were presentative of five independent experiments. Values were shown as means± S.E.M. $^{##}P < 0.01$ *v.s.* Normal group. $^{**}P < 0.01$ *v.s.* LPS + ATP group.

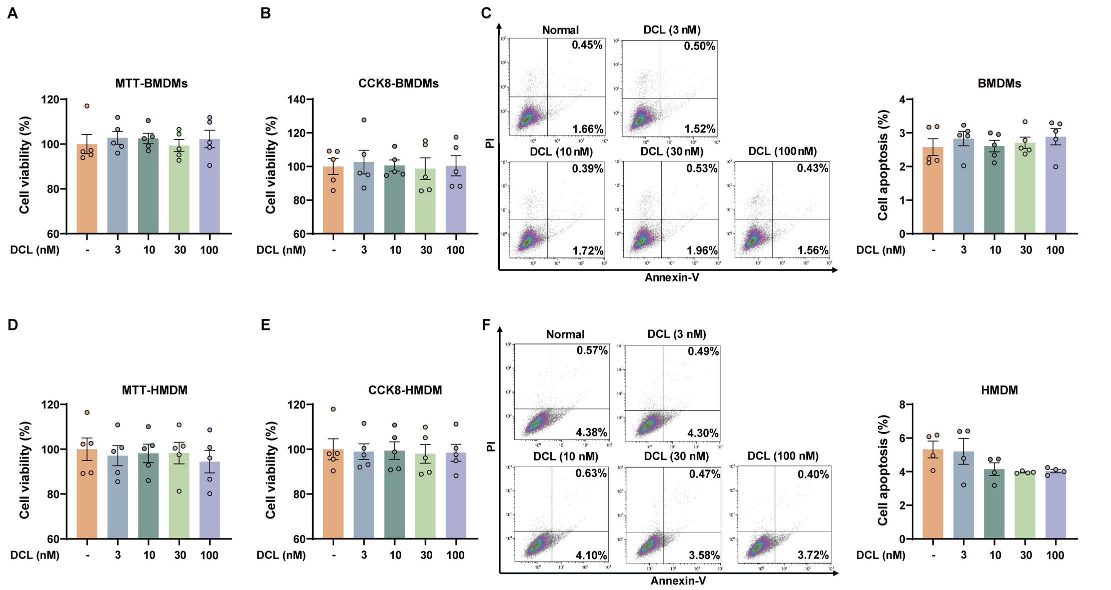

Supplementary Figure 2. DCL exerted little effect on macrophages. BMDMs were treated with DCL (3, 10, 30, 100 nM) for 24 h, the cell viability and apoptosis were determined by MTT (A, D), CCK8 (B, E) and flow cytometry (C, F), respectively. Data from *in vitro* assays were presentative of five independent experiments. Values were shown as means± S.E.M.

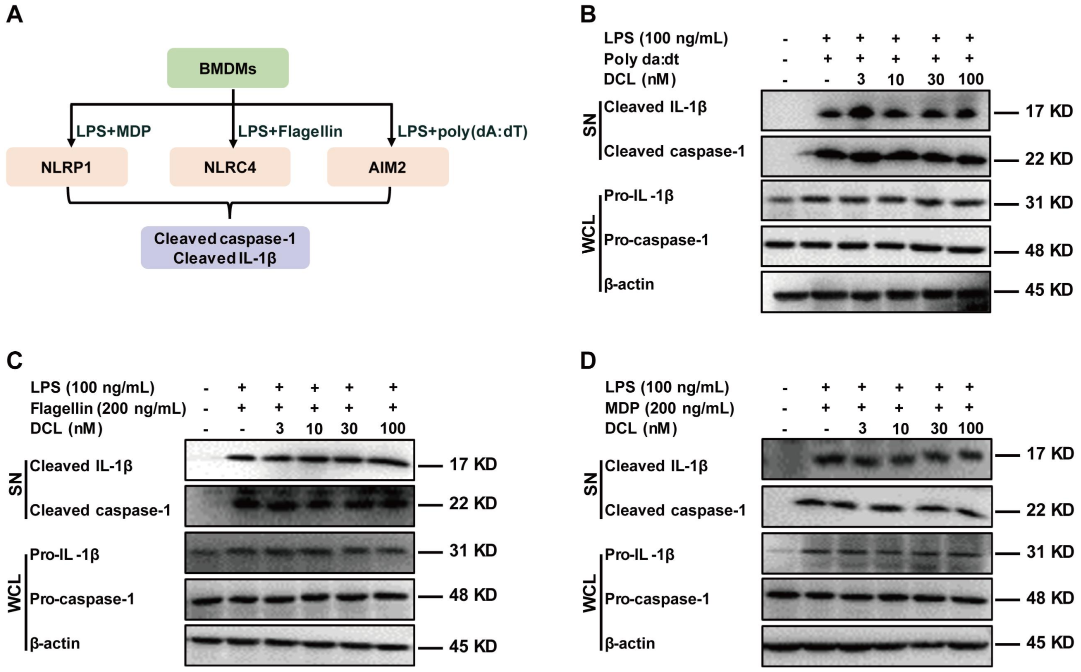

Supplementary Figure 3. DCL has minor effect on AIM2, NLRC4 or NLRP1 inflammasome activation. (A-C) BMDMs were subjected to the following treatment: LPS priming (100 ng/mL, 4 h), DCL (3, 10, 30, 100 nM, 1 h) and poly (dA: dT), MDP and flagellin. Western blotting analysis of cleaved caspase-1 and cleaved IL-1β. Data from *in vitro* assays were presentative of five independent experiments. Values were shown as means± S.E.M.

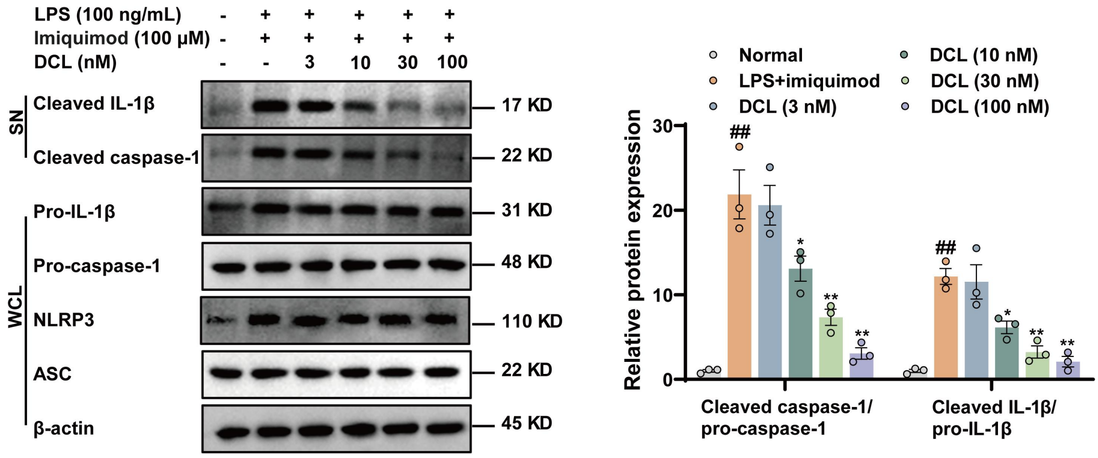

Supplementary Figure 4. DCL-inhibited NLRP3 inflammasome activation was independent of K^+ efflux. The BMDMs were primed with LPS (100 ng/mL) for 4 hours, followed by incubation with DCL (3, 10, 30, 100 nM) for 1 h. Subsequently, the cells were treated with imiquimod (100 μM) for 2 h. the protein expressions of cleaved caspase-1 and cleaved IL-1β in supernatant were detected by western blotting assay. Data from *in vitro* assays were presentative of five independent experiments. Values were shown as means± S.E.M. $^{\#\#}P < 0.01$ *v.s.* Normal group. $^{**}P < 0.01$ *v.s.* LPS + imiquimod group.

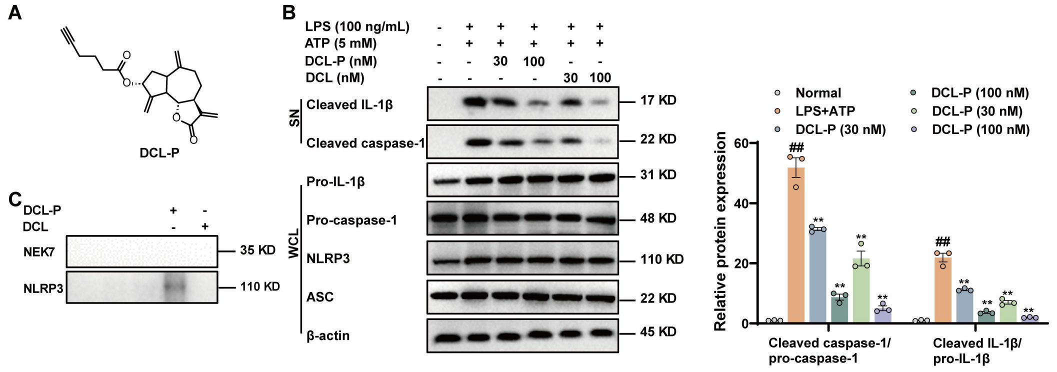

Supplementary Figure 5. DCL-P significantly inhibited NLRP3 inflammasome activation. (A) The chemical structure of DCL-P. (B) The BMDMs were primed with LPS (100 ng/mL) for 4 hours, followed by incubation with DCL-P (30, 100 nM) and DCL (30, 100 nM) for 1 h. Subsequently, the cells were treated with ATP (5 mM) for 2 h. the protein expressions of cleaved caspase-1 and cleaved IL-1β in supernatant were detected by western blotting assay. (C) The biotinylated DCL-P was incubated with lysates prepared from BMDMs transfected with NLRP3 or NEK7 plasmids. Cell lysates were prepared using NP-40 lysis buffer and incubated with streptavidin-conjugated magnetic beads at 4 °C for 4 hours. The protein expressions of NLPR3 and NEK7 were assessed by western blotting assay. Data from *in vitro* assays were presentative of five independent experiments. Values were shown as means± S.E.M. $^{\#\#}P < 0.01$ *v.s.* Normal group. $^{**}P < 0.01$ *v.s.* LPS + ATP group.

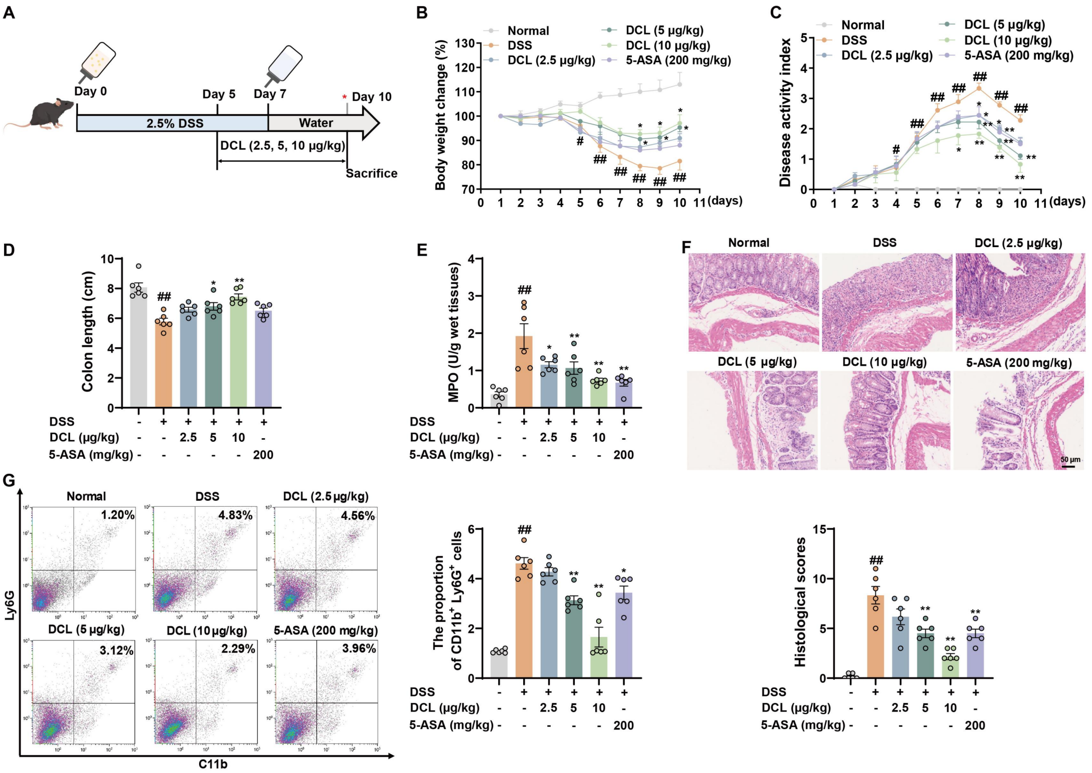

Supplementary Figure 6. DCL possesses therapeutic effect on DSS-induced colitis in mice. Mice were subjected to DSS-induced colitis. DCL (2.5, 5, 10 μg/kg) and 5-ASA (200 mg/kg) were given to the mice from day 5 to day 10 (A). (B) Body weight reduction rate of mice. (C) The disease activity index (DAI) scores were calculated. (D) Colon length during DSS challenge. (E) The myeloperoxidase (MPO) activity was assessed with kits. (F) Hematoxylin and eosin-stained slides from the same parts of colons in mice. (G) The infiltration of neutrophils in colonic lamina propria was assessed by flow cytometry assay. Data were presentative of six mice in each group and shown as means± S.E.M. $^{\#}P < 0.05$, $^{\#\#}P < 0.01$ *v.s.* Normal group. $^{*}P < 0.05$, $^{**}P < 0.01$ *v.s.* DSS group.

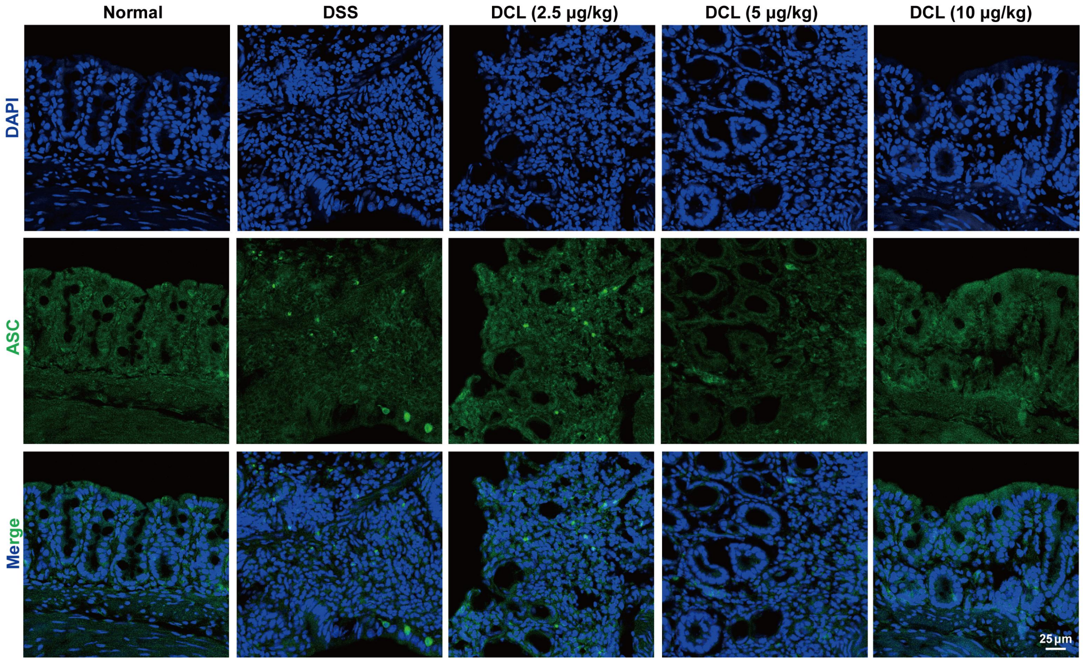

Supplementary Figure 7. DCL significantly decreases ASC puncta formation in colitis mice. The mice were subjected to DSS-induced colitis model, and DCL was injected intraperitoneally for 10 consecutive days. The ASC speck formation in colonic tissues was detected by immunofluorescence assay.

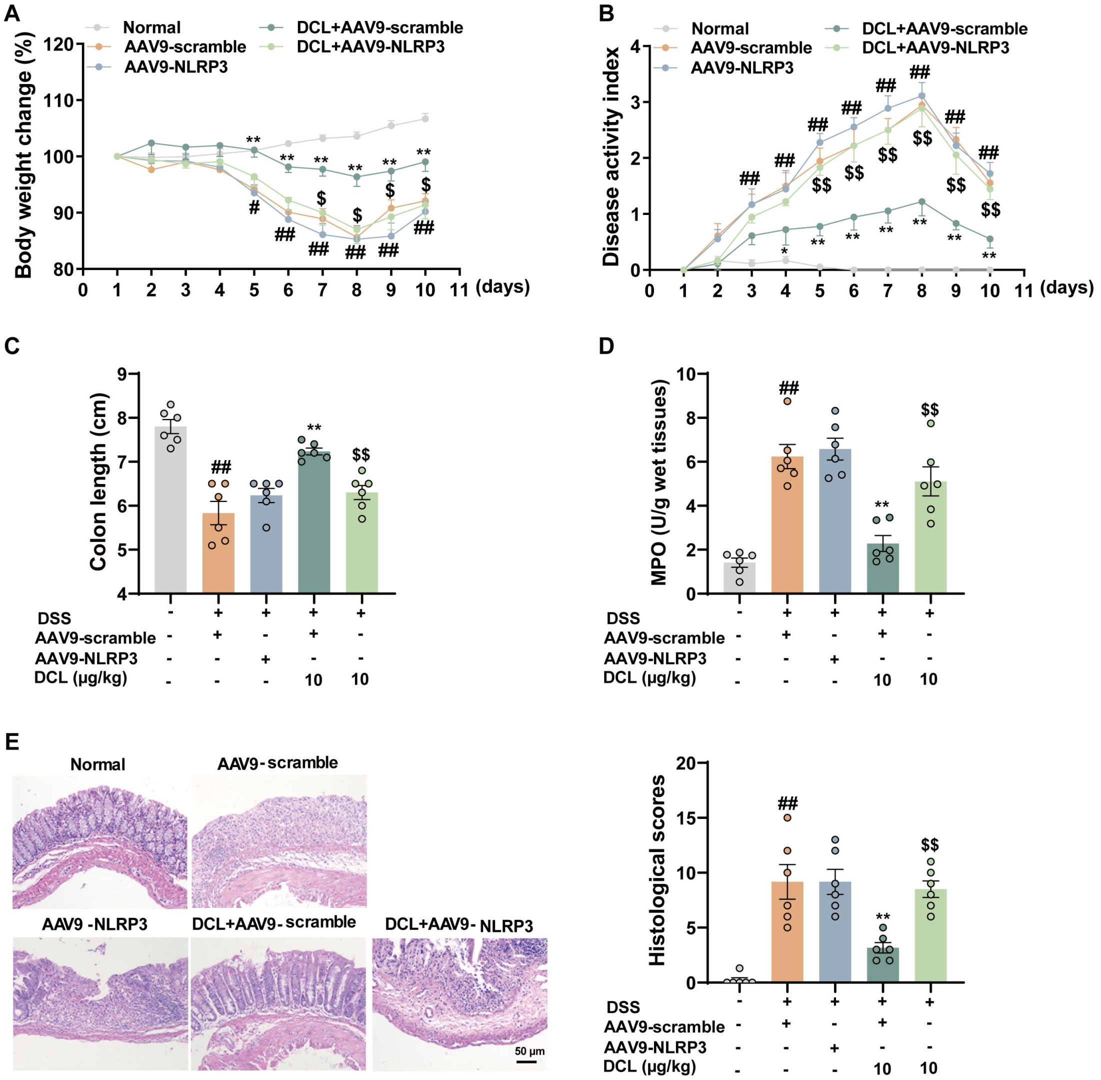

Supplementary Figure 8. DCL targets NLRP3 inflammasome activation in macrophages to alleviate colitis. The mice were rectally injected NLRP3 highly expressed adeno-associated virus 9 carrying F4/80 in the promoter region. Four week later, the mice were subjected to DSS-induced colitis model, and DCL was injected intraperitoneally for 10 consecutive days. (A) Body weight reduction rate of mice. (B) The disease activity index (DAI) scores were calculated. (C) Colon length during DSS challenge. (D) The myeloperoxidase (MPO) activity was assessed with kits. (E) Hematoxylin and eosin-stained slides from the same parts of colons in mice. Data were presentative of at least of six mice in each group and shown as means± S.E.M. $^{\#}P < 0.05$, $^{\#\#}P < 0.01$ *v.s.* Normal group. $^{*}P < 0.05$, $^{**}P < 0.01$ *v.s.* DSS+AAV9-scramble group. $^{\$}P < 0.05$, $^{\$\$}P < 0.01$ *v.s.* DCL+AAV9-scramble group.

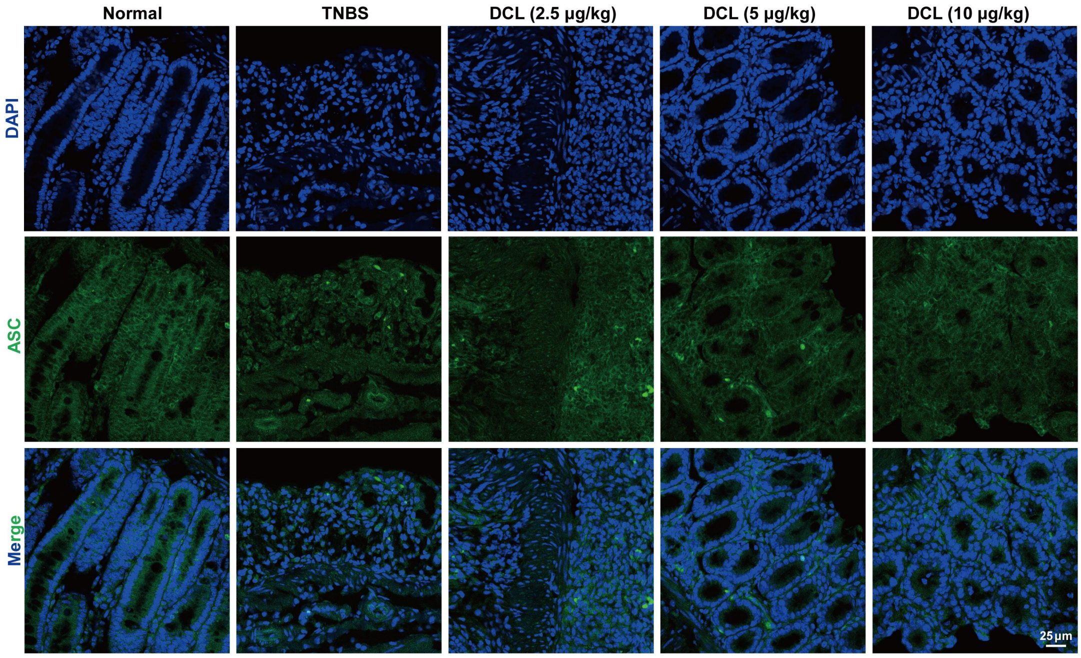

Supplementary Figure 9. DCL significantly decreases ASC puncta formation in the mice with Chron's disease. The mice were subjected to TNBS-induced Chron's disease model, and DCL was injected intraperitoneally for 7 consecutive days. The ASC speck formation in colonic tissues was detected by immunofluorescence assay.

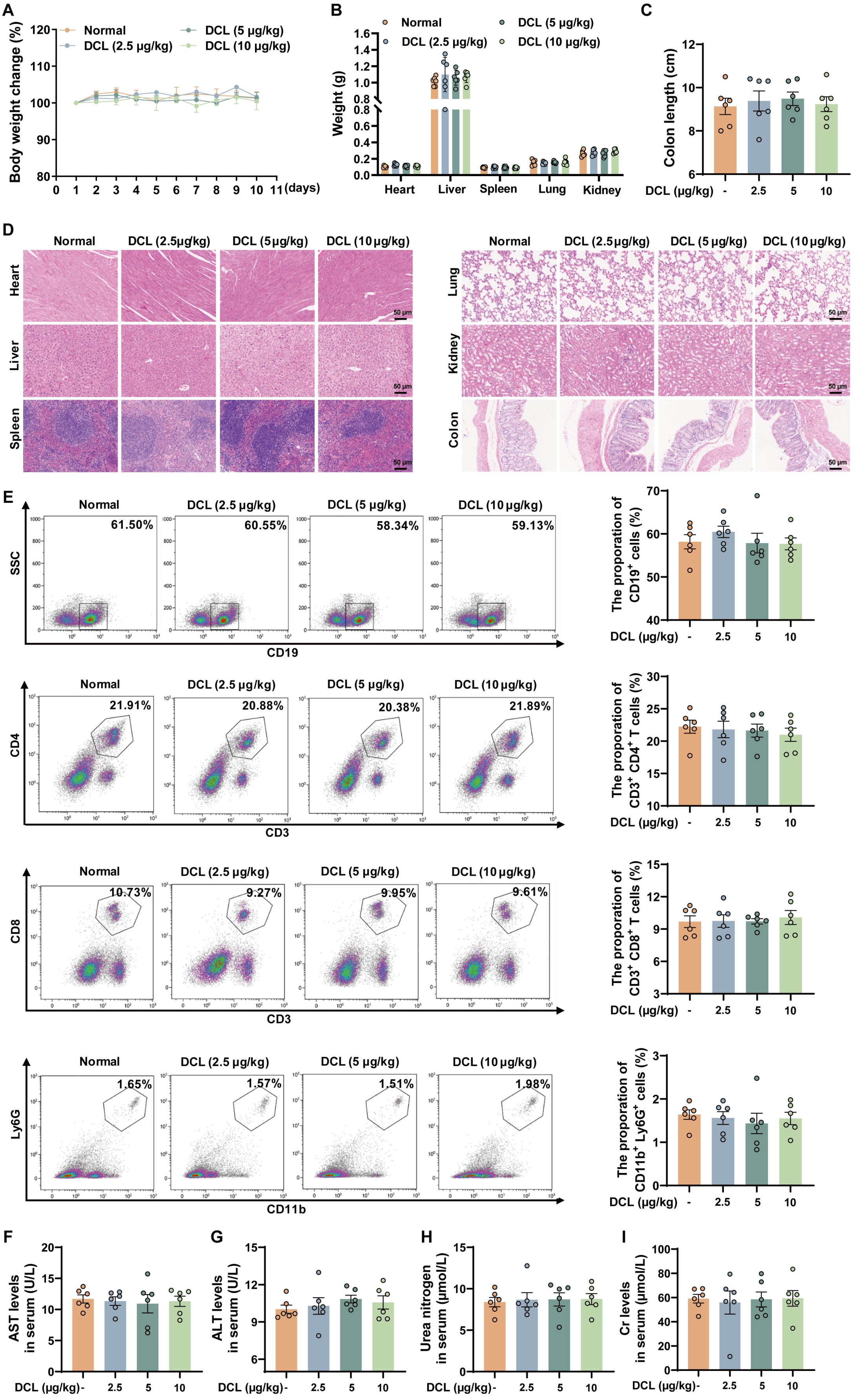

Supplementary Figure 10. DCL has minor effect on health mice. Mice were

injected intraperitoneally with DCL (2.5, 5, 10 μg/kg) daily for 10 days. (A) Body weight loss. (B) Organ weight. (C) Colon length. (D) Hematoxylin and eosin-stained slides of heart, liver, spleen, lung, kidney in mice. (E) Proportion of $CD19^+$ B cells, $CD3^+$ $CD4^+$ T cells, $CD3^+$ $CD8^+$ T cells, $CD11b^+$ $Ly6G^+$ cells in spleen of mice. (F-I) The serum levels of aspartate aminotransferase (AST), alanine aminotransferase (ALT), creatinine and urea nitrogen were detected by kits. Data were presentative of at least of six mice in each group and shown as means± S.E.M.

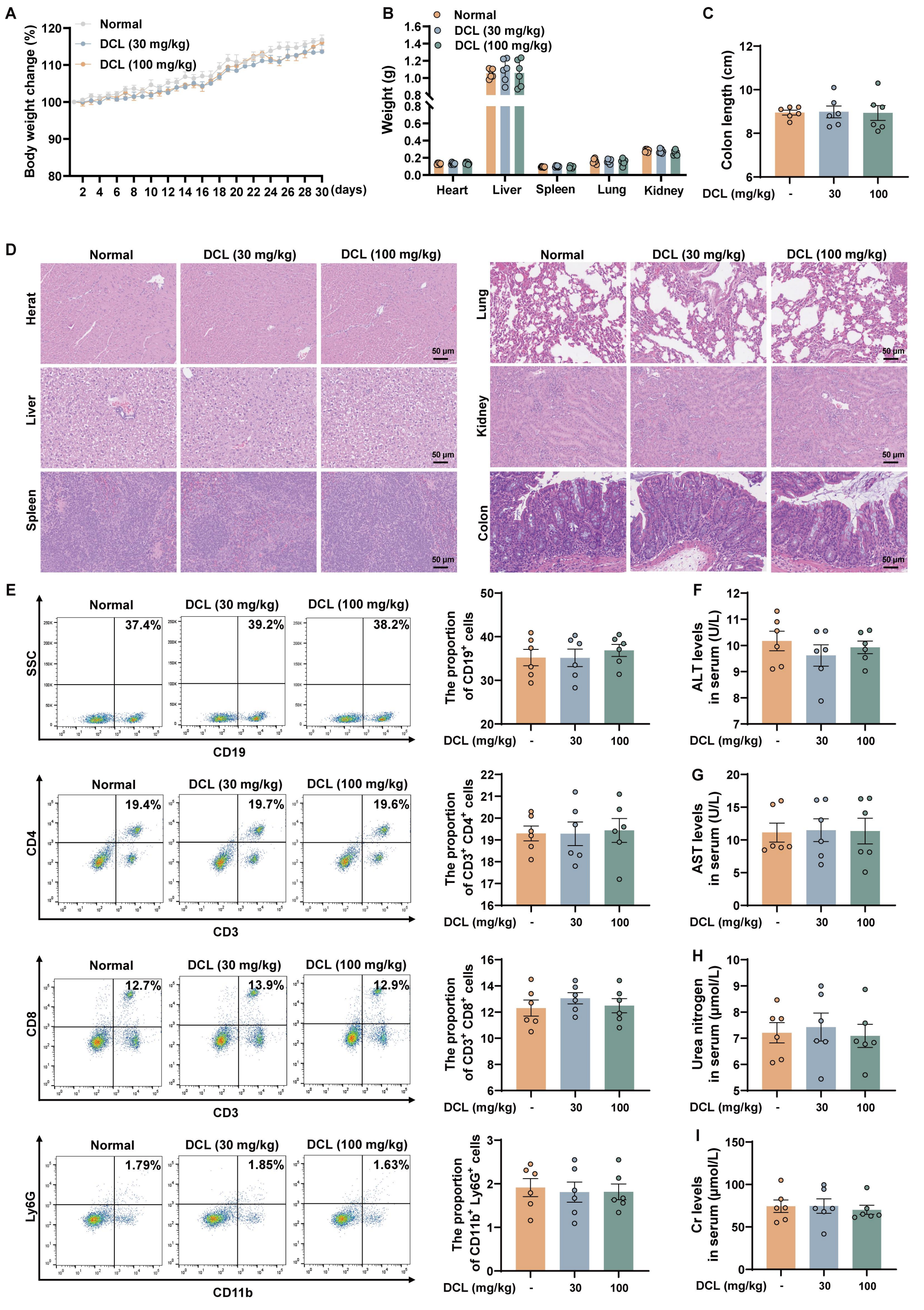

Supplementary Figure 11. DCL possesses little effect on health mice. Mice were injected intraperitoneally with DCL (30, 100 mg/kg) daily for 30 days. (A) Body weight loss. (B) Organ weight. (C) Colon length. (D) Hematoxylin and eosin-stained slides of heart, liver, spleen, lung, kidney in mice. (E) Proportion of $CD19^+$ B cells,

$CD3^+$ $CD4^+$ T cells, $CD3^+$ $CD8^+$ T cells, $CD11b^+$ $Ly6G^+$ cells in spleen of mice. (F-I) The serum levels of aspartate aminotransferase (AST), alanine aminotransferase (ALT), creatinine and urea nitrogen were detected by kits. Data were presentative of at least of six mice in each group and shown as means± S.E.M.
